# Efficacy of an emotion-oriented cognitive behavior therapy for delusions (CBTd-E) compared to waitlist in a single-blinded randomized-controlled trial

**DOI:** 10.1038/s41537-026-00737-y

**Published:** 2026-02-17

**Authors:** Stephanie Mehl, Christopher Hautmann, Björn Schlier, Laura Marie-Louise Dorn, Lea Ludwig, Tobias Teismann, Michael Franz, Winfried Rief, Rudolf Stark, Tania Marie Lincoln

**Affiliations:** 1 Marburg University, Department of Psychiatry and Psychotherapy, Marburg, Germany; 2 Department of Health and Social Work, Frankfurt University of Applied Sciences, Frankfurt am Main, Germany; 3 Department of Child and Adolescent Psychiatry, Psychosomatics and Psychotherapy, Faculty of Medicine and University Hospital of Cologne, Köln, Germany; 4 Department of Psychology, University of Wuppertal, Wuppertal, Germany; 5 Department of Clinical Psychology and Psychotherapy, University of Hamburg, Hamburg, Germany; 6 Marburg University, Department of Clinical Psychology and Psychotherapy, Marburg, Germany; 7 Mental Health Research and Treatment Center, Ruhr-University Bochum, Bochum, Germany; 8 Department of Psychiatry and Psychotherapy, Justus-Liebig University of Gießen, Gießen, Germany; 9 Vitos Clinic for Psychiatry and Psychotherapy, Gießen & Marburg, Marburg, Germany; 10 Department of Psychotherapy and System Neuroscience, Justus-Liebig University of Gießen, Gießen, Germany

**Keywords:** Human behaviour, Psychosis, Schizophrenia

## Abstract

Psychological interventions for delusions may be enhanced by targeting their presumed causal factors. An emotion-oriented variant of cognitive behavioral therapy for delusions (CBTd-E), designed to target affect regulation and maladaptive schemata, was evaluated for its effect on delusions. A single-blind, multicenter, randomized, waitlist-controlled trial was conducted in three German outpatient clinics. Ninety-four patients with psychotic disorders and persistent delusions were randomized to 25 individual sessions of CBTd-E over 6 months (*n* = 47) or waitlist (*n* = 47). CBTd-E included two modules designed to improve affect regulation and maladaptive schemata. Assessments were performed at baseline (T1), three months (T2), and six months (T3). Regression-based analysis of covariance at T3 in the intent-to-treat sample indicated no significant benefit for the CBT-E group in the primary outcome (Psychotic Symptom Rating Scale delusions subscale, *d* = -0.45 [CI: 0.36; -1.26]). Regarding secondary outcomes, a significant effect favoring CBTd-E was observed in general psychopathology (*d* = -0.56), but no effects on positive and negative symptoms, depression, general and social functioning, or antipsychotic dosage. Regarding the proposed target mechanisms, we found improved cognitive reappraisal (*d* = 0.59), worrying (*d* = -0.52), quality of sleep (*d* = -0.49), and self-esteem (*d* = 0.36). Despite its effect on the suggested target mechanisms, affect regulation and maladaptive schemata, and on general psychopathology, this emotion-focused variant of CBT did not show an effect on delusions. A possible avenue to achieve stronger effects on delusions is to personalize the modularized interventions.

**Trial registration**: Clinicaltrials.gov Identifier: NCT02787135

## Introduction

Cognitive Behavior Therapy (CBTp) for patients with positive symptoms of psychosis has been successful in improving positive symptoms, general psychopathology, and relapse prevention^1–3^. However, CBTp’s effect on delusional beliefs is small compared to routine treatment and is not superior to other psychological interventions^4,5^. Several researchers have aimed to improve CBTp’s effectiveness for delusions by using ‘causal interventionist’ approaches^6^ that target specific causal factors related to the delusion(s) and focus on these alone^7–10^ rather than engaging in examining and testing delusional beliefs^11^.

Based on theoretical models that assume a *cognitive* and *affective pathway* to be involved in the development of delusions^12–15^, ‘targeted’ interventions can be broadly categorized into interventions that aim to improve *cognitive factors* and interventions that aim to improve *affective factors*.

Regarding the *cognitive pathway*, Garety and colleagues developed the ‘Slow Mo’ intervention that aims to improve the jumping-to-conclusions (JTC) bias and belief flexibility. Their approach was superior to routine care in improving delusions and the targeted cognitive factors in two randomized controlled trials (RCTs)^16,17^. Another approach is the Metacognitive Training^18^, which has also been shown to be effective in improving both the targeted cognitive factors and delusions in numerous studies^19^. Regarding the *affective pathway*, Freeman and colleagues found that brief interventions that focused on worry^8,10^ significantly improved both worry and delusional conviction/distress compared to routine care. Interventions focusing on self-esteem^7^ or quality of sleep^9^ also improved the intervention targets, but their effects on delusions were less clear.

Given the limited research on interventions that address the affective pathway and considering patients’ preference for interventions that focus on emotions^20,21^, it could be promising to further develop interventions that target affective mechanisms.

The present study aims to test the potential of targeting the full affective pathway postulated in models of delusions^12–15,22^, improving both affective states and associated negative self-schemata as targets. The approach is based on strong evidence linking these factors to delusions. For instance, several studies link emotional instability in daily life to persecutory delusions^23–25^ and show increases in negative affect and arousal (i.e., unregulated or insufficiently regulated emotions) to precede increases in delusions^26–29^. There is also clear evidence for pronounced difficulties in emotion awareness and regulation^25,27,30,31^ and the ability to maintain healthy sleep patterns^32–35^ that have been found to contribute to affective instability and delusions in daily life. Targeting emotion regulation (ER) in a broader sense^36^ aligns well with this evidence base.

The relevance of negative self-schemata to delusions is also well backed up by ecological momentary assessment (EMA) and longitudinal studies showing decreases in self-esteem to precede increases in persecutory delusions^37–39^ or positive symptoms of psychosis more generally^40,41^ over time. In addition, cross-sectional questionnaire research consistently shows a strong association between low self-esteem and paranoia^42,43^ and positive symptoms of psychosis^44–46^.

Building on this research, the present study tests a cognitive behavioral intervention that focuses on the affective pathway to delusions. The intervention addresses emotion awareness, adaptive and maladaptive ways of regulating different types of emotions, and behavior relevant to maintaining emotional stability (which will be referred to as affect regulation), as well as negative schemata related to oneself and others, self-esteem, and self-acceptance (which will be referred to as maladaptive schemata). The first version of the intervention was piloted in 25 sessions over a six-month period, where it proved feasible and acceptable^47^. As the pilot RCT did not produce sufficiently strong effects on the target mechanisms, we modified the manual based on feedback from patients and therapists. Modifications included a stronger focus on testing ER skills in daily life, including interventions focused on sleep quality into the module affect regulation, intensified therapist training and supervision, and adherence monitoring.

Here, we investigate whether the modified intervention is more effective than routine care (waitlist) in (1) reducing delusions (primary outcome), (2) improving positive symptoms, negative symptoms, general psychopathology, depression, general and social functioning, and resulting in lower dosages of antipsychotic medication at 6-months post-assessment (secondary outcomes). In order to judge whether the intervention worked as intended, we will also report its effects on emotional stability and ability to regulate one’s own emotions, quality of sleep, and self-esteem at 6-months post-assessment.

## Results

### Flow of participants and adverse events

**Fig. 1:**
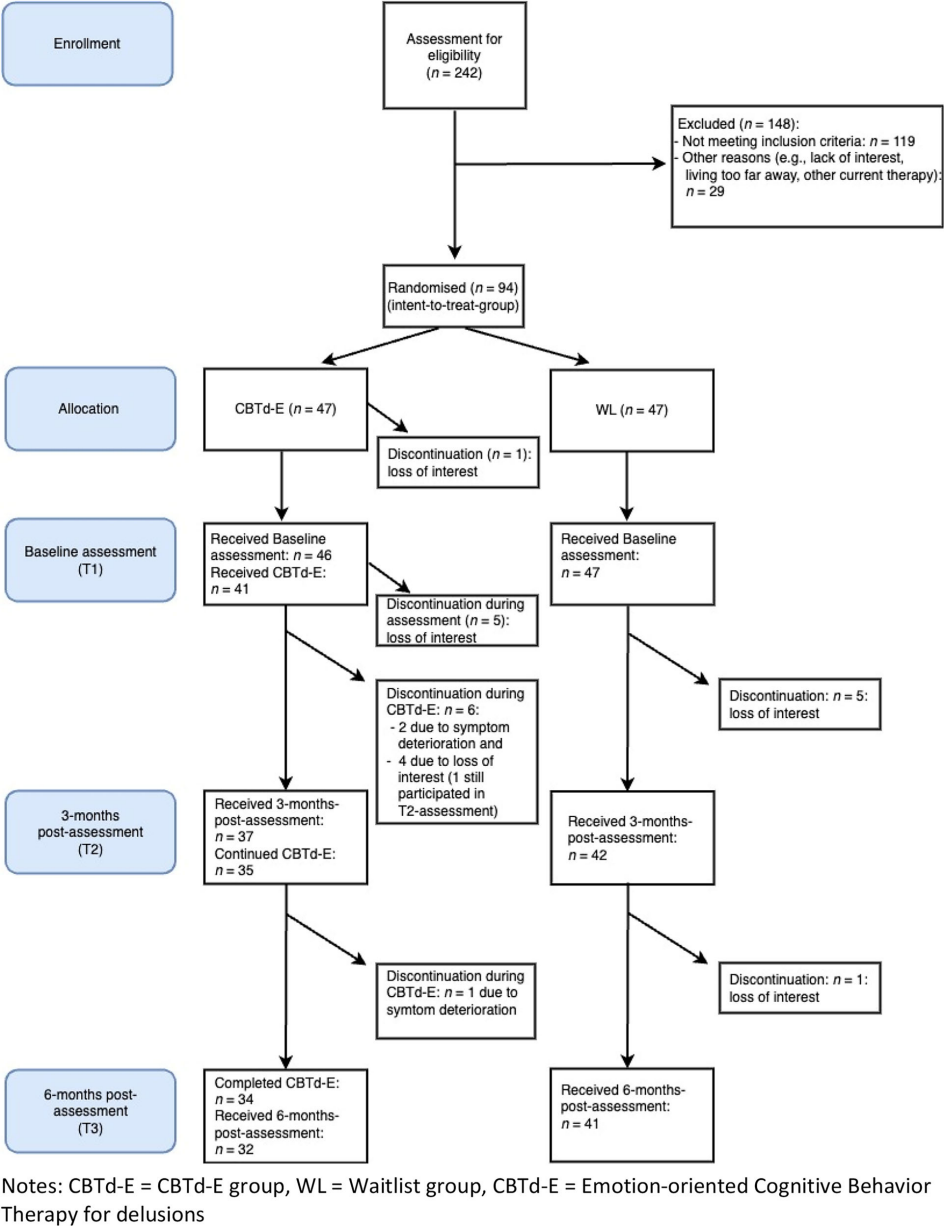
Flowchart of the study.

The number of recruited, screened, and randomized patients is depicted in Fig. 1. Feasibility and acceptance indicators are as follows: 94 patients signed informed consent and were randomized (93.07% of all eligible patients). They are referred to as the Intent-to-Treat (ITT) sample. Most patients in the CBTd-E group completed the full CBTd-E intervention over 6 months (*n* = 34, 72.34%). As reported in Fig. 1, 13 patients dropped out during the intervention (27.66%). The causes for drop-out were loss of interest in CBTd-E (6 during diagnostic assessment, four during the first 3 months of CBTd-E) and symptom deterioration (2 in the first three months, 1 in the last three months). In the WL group, five patients dropped out at (or were unreachable for) 3-months post-assessment (T2) and one patient at 6-months post-assessment (T3).

**Table 1:** Sociodemographic and clinical characteristics of patients with psychotic disorders enrolled in the study and allocated to the CBTd-E group or the Waitlist group (Intention-to-Treat Sample; *n* = 94).

Variable	CBTd-E group (*n* = 47) *M (SD)/ n (%)*	WL group (*n* = 47) *M (SD)/ n (%)*
*Sociodemographic variables*		
Age (years)	37.85 (11.09)	35.72 (12.59)
Sex assigned at birth		
Female	16 (34.04%)	20 (42.55%)
Male	31 (65.96%)	27 (57.45%)
Education grade^a^		
No graduation:	0 (0%)	1 (2.13%)
9 school years ^a^:	9 (19.15%)	12 (25.53%)
10 school years ^b^:	10 (21.28%)	13 (27.66%)
High school equivalent:	28 (59.57%)	21 (44.68%)
Estimated verbal IQ	104.68 (13.3)^c^	99.5 (12.42) ^d^
Family status:		
Single	37 (78.72%)	29 (61.71%)
In relationship	4 (8.51%)	9 (19.14%)
Married	5 (10.64%)	5 (10.64%)
Divorced	1 (2.13%)	4 (8.51%)
Living situation:		
Living alone:	22 (46.81%)	18 (38.30%)
Living with partner:	8 (17.02%)	6 (12.77%)
Living with parents:	5 (10.64%)	7 (14.89%)
Shared flat:	5 (10.64%)	6 (12.77%)
Assisted living:	7 (14.89%)	9 (19.14%)
Homeless:		1 (2.13%)
*Clinical variables*		
Main diagnosis:		
Schizophrenia	36 (76.59%)	32 (68.08%)
Schizoaffective disorder	7 (14.89%)	10 (21.28%)
Delusional disorder	1 (2.13%)	5 (10.64%)
Schizotypal personality disorder	2 (4.26%)	0 (0%)
Brief psychotic disorder	1 (2.13%)	0 (0%)
Medication at baseline:	43 (91.49%)	42 (86.36%)
Antipsychotic medication	41 (87.23%)	41 (87.23%)
Antidepressive medication	10 (21.28%)	11 (23.4%)
Duration of psychotic disorder	14.66 (11.47)	11.66 (9.64%)
PANSS P1 delusions	4.09 (1.19)	4.13 (1.00)
Acute delusions	47 (100%)	47 (100%)
Persecutory delusions	33 (70.21%)	38 (80.84%)
Delusions of grandeur	1 (2.13%)	1 (2.13%)
Delusions of guilt	4 (8.51%)	0 (0%)
Somatic delusions	0 (0%)	2 (4.26%)
Religious delusions	2 (4.26%)	1 (2.13%)
Delusions of love	7 (14.89%)	3 (6.38%)
Other delusions	0 (0%)	2 (4.26%)
PSYRATS subscale delusion conviction: score of at least 3 ^e^	31 (67.39%)	34 (72.34%)

*WL* Waitlist group, *M* mean, *SD* standard deviation.

^a^German Hauptschulabschluss (9 school years).

^b^German Realschulabschluss (10 school years).

^c^*n* = 37.

^d^*n* = 34.

^e^Inclusion criteria of two targeted studies (Freeman et al.^7^; Freeman et al.^8,9^).

There was no statistically significant group difference in the number of drop-outs regarding the baseline assessment (T1: CBTd-E: *n* = 1 (2.1%); WL: *n* = 0 (0%); χ^2^ (1) = 1.01; *p* = .315), at T2 assessment (CBTd-E: *n* = 12 (25.5%); WL: *n* = 5 (10.6%); χ^2^ (1) = 3.52; *p* = .106) or at T3 assessment (CBTd-E: *n* = 13 (27.7%); WL: *n* = 6 (12.8%); χ^2^ (1) = 3.23; *p* = .122).

A comparison of completers (*n* = 34) and patients who dropped out during CBTd-E (*n* = 13) revealed no statistically significant differences in sociodemographic, clinical, or any of the outcome variables (Supplement Tables S2 and S3).

Eight serious adverse events occurred. Three patients in the CBTd-E group experienced symptom deterioration and were hospitalized; five patients in the WL group experienced symptom deterioration, and three of them were hospitalized. Patients in the intervention group continued the intervention after the hospital stay. The PIs responsible for each center (TL, SM, TT) were responsible for classifying serious adverse events. No serious adverse events were classified as related to the trial intervention or procedures.

Table 1 presents the sociodemographic and clinical variables in the intent-to-treat sample. Table 2 presents the means and standard deviations for the primary and secondary outcome variables at all three assessment time points.

**Table 2:** Mean and standard deviations of the CBTd-E group and the Waitlist group at baseline, 3-months post-assessment, and 6-months post-assessment.

	CBTd-E group baseline (T1)		WL group baseline (T1)		CBTd-E group 3-months post-assessment (T2)		WL group 3-months post-assessment (T2)		CBTd-E group 6-months post-assessment (T3)		WL group 6-months post-assessment (T3)	
Measure	Mean (SD)	*n*	Mean (SD)	*n*	Mean (SD)	*n*	Mean (SD)	*n*	Mean (SD)	*n*	Mean (SD)	*n*
*Primary outcome variables*												
PSYRATS delusions scale (Blind)	12.58 (5.41)	33	14.79 (3.57)	28	10.45 (5.76)	31	12.21 (5.86)	29	8.73 (6.47)	22	11.41 (5.95)	27
PSYRATS delusions scale (Unblinded)	14.93 (3.56)	46	15.34 (3.50)	47	9.32 (6.89)	37	11.00 (7.40)	42	9.47 (6.25)	32	11.66 (6.77)	41
*Secondary outcome variables*												
PDI-21 grand total score (SR)	68.50 (56.15)	44	76.25 (58.03)	44	56.48 (58.46)	31	73.97 (67.98)	38	44.57 (46.30)	31	58.57 (56.98)	37
EMA persecutory delusions (SR)	2.52 (1.35)	32	2.77 (1.44)	40	2.73 (1.50)	25	2.50 (1.46)	27	2.61 (1.46)	24	2.64 (1.33)	25
PANSS POS (Blind)	15.94 (4.06)	32	16.87 (4.92)	30	-	-	-	-	14.55 (5.18)	22	16.07 (5.16)	29
PANSS POS (Unblinded)	16.84 (3.34)	46	16.73 (4.13)	47	-	-	-	-	13.61 (4.74)	31	15.31 (4.64)	39
PANSS NEG (Blind)	16.47 (3.73)	32	15.90 (5.34)	30	-	-	-	-	14.82 (5.10)	22	13.93 (4.71)	29
PANSS NEG (Unblinded)	15.83 (4.76)	46	15.59 (4.83)	47	-	-	-	-	14.48 (5.30)	31	14.44 (5.19)	39
PANSS GEN (Blind)	36.13 (6.59)	32	36.53 (6.97)	30	-	-	-	-	31.50 (8.09)	22	34.21 (7.68)	29
PANSS GEN (Unblinded)	33.50 (6.77)	46	34.00 (6.18)	47	-	-	-	-	28.94 (8.19)	31	32.18 (7.32)	39
CDSS total score (Blind)	6.50 (3.89)	32	5.67 (3.47)	30	-	-	-	-	4.56 (4.09)	22	5.21 (3.89)	29
CDSS total score (Unblinded)	6.98 (4.61)	46	5.91 (3.88)	47	-	-	-	-	4.13 (3.75)	31	5.68 (3.92)	38
RFS general functioning (Blind)	7.00 (2.07)	32	7.63 (2.30)	29	-	-	-	-	6.98 (2.85)	22	7.68 (2.56)	28
RFS general functioning (Unblinded)	6.48 (2.34)	46	6.39 (2.76)	47	-	-	-	-	7.77 (2.73)	31	6.88 (2.85)	37
RFS social functioning (Blind)	7.45 (2.22)	32	7.02 (2.16)	29	-	-	-	-	8.05 (2.60)	22	7.22 (2.98)	28
RFS social functioning (Unblinded)	6.76 (2.30)	46	6.35 (2.37)	47	-	-	-	-	7.77 (2.94)	31	6.50 (2.92)	37
CPZ	661.74 (475.37)	46	588.53 (529.81)	47	604.08 (469.49)	36	516.72 (503.65)	42	594.62 (446.12)	30	646.56 (882.57)	38
PSYRATS voices scale (Blind)	8.94 (13.17)	33	7.79 (10.38)	28	11.03 (13.15)	31	5.93 (9.97)	29	8.36 (13.06)	22	8.22 (12.67)	27
PSYRATS voices scale (Unblinded)	11.13 (14.30)	46	9.17 (12.40)	47	9.92 (12.37)	37	7.83 (12.33)	42	8.06 (11.98)	32	9.39 (13.12)	41
*Target mechanisms*												
ERQ expressive suppression (SR)	13.91 (5.01)	43	12.10 (5.79)	45	13.93 (6.21)	30	10.25 (5.41)	38	12.57 (5.45)	28	10.56 (4.51)	36
ERQ reappraisal (SR)	16.00 (6.63)	43	17.14 (8.19)	44	20.43 (7.02)	30	16.06 (8.51)	37	19.64 (5.47)	28	16.73 (6.56)	37
ERSQ total score (SR)	52.86 (19.01)	43	54.60 (14.43)	45	52.96 (19.66)	31	56.36 (18.21)	38	58.74 (21.75)	28	59.95 (17.99)	36
ERI positive emotions (SR)	22.11 (10.39)	44	23.42 (9.88)	45	22.29 (10.53)	31	25.63 (9.24)	38	21.69 (10.30)	28	26.70 (10.84)	37
ERI negative emotions (SR)	39.96 (11.89)	44	40.30 (10.04)	45	37.96 (12.52)	31	37.56 (11.85)	38	38.86 (13.06)	28	42.05 (11.66)	37
PSWQ total sum score (SR)	53.09 (13.20)	44	53.16 (12.13)	45	45.79 (17.62)	30	55.88 (15.66)	38	45.02 (14.76)	28	50.12 (14.80)	37
ISI insomnia sum score (SR)	11.98 (6.14)	43	13.11 (6.35)	44	9.93 (6.48)	30	13.73 (6.44)	38	9.19 (6.16)	28	13.22 (6.23)	37
RSES sum score (SR)	15.82 (7.06)	44	15.29 (6.84)	45	17.93 (7.22)	31	15.94 (6.72)	38	19.64 (6.51)	28	16.87 (6.85)	37
SCS total score (SR)	47.29 (14.30)	44	45.57 (17.36)	44	51.02 (17.84)	31	44.97 (15.72)	37	53.02 (15.85)	28	50.25 (16.63)	37
BCSS negative-self (SR)	9.13 (5.79)	43	10.02 (5.57)	45	8.93 (6.18)	30	9.05 (5.38)	37	7.25 (5.78)	28	8.33 (5.13)	35
BCSS positive-self (SR)	12.23 (5.04)	43	10.13 (5.53)	45	12.73 (5.44)	30	12.14 (5.86)	37	13.57 (5.83)	28	11.97 (4.88)	35
BCSS negative-other (SR)	9.31 (5.68)	43	10.52 (5.89)	44	8.10 (6.10)	30	9.84 (5.78)	37	7.31 (5.59)	28	9.06 (5.85)	36
BCSS positive-other (SR)	12.64 (4.47)	43	11.24 (4.15)	45	13.54 (4.14)	30	12.15 (5.68)	37	12.55 (5.27)	28	11.83 (4.59)	36

*WL* Waitlist, *SD* standard deviation, *SR* Self-rating, *PSYRATS* psychotic rating symptom scale, *PDI* Peters et al. delusions inventory, *EMA* ecological momentary assessment of persecutory delusions over 6 consecutive days and 10 time points: mean score, *PANSS* positive and negative syndrome scale, *PANSS POS* PANSS positive scale, *PANSS NEG* PANSS negative scale, *PANSS GEN* PANSS general psychopathology scale, *CDSS* Calgary depression rating scale, *RFS* role functioning scale, *CPZ* chlorpromazine equivalent of antipsychotic medication, *ERQ* emotion regulation questionnaire, *ERSQ* emotion regulation style questionnaire, *ERI* emotion regulation inventory, *PSWQ* Penn State worry questionnaire, *ISI* insomnia severity index, *RSE* Rosenberg self-esteem scale, *SCS* self-compassion scale, *BCSS* brief core schema scale.

### Change in primary and secondary outcome criteria

Results from the regression-based ANCOVA using FIML for handling missing data during the 3-month assessment (T2) are shown in Table 3. Participants received the module on *affect regulation* before this assessment. No statistically significant group effects were found for delusion outcomes (PSYRATS delusions subscale: blinded and unblinded assessments). Additionally, no statistically significant effects favoring CBTd-E across any of the secondary outcome variables were found.

**Table 3:** Results of the regression-based ANCOVA with Full Information Maximum Likelihood (FIML) for missing data handling at 3-months post-assessment (T2) on the primary and secondary outcome variables in the intent-to-treat sample (ITT: *n* = 94).

Measure	*n (analysis)*	Group differences *beta*_unst_.^a^*SE*		*p*	Effect size *d 95% CI*	
*Primary outcome variables*						
PSYRATS delusions scale (Blind)	61	−1.423	1.368	0.298	−0.300	[−0.864; 0.265]
PSYRATS delusions scale (Unblinded)	93	−1.665	1.388	0.230	−0.473	[−1.246; 0.300]
*Secondary outcome variables*						
PDI-21 grand total score (SR)	88	−6.106	9.527	0.522	−0.107	[−0.435; 0.221]
EMA persecutory delusions (SR)	72	0.295	0.265	0.265	0.212	[−0.160; 0.584]
CPZ	93	6.860	55.168	0.901	0.014	[−0.202; 0.229]
PSYRATS voices scale (Blind)	61	2.240	1.368	0.102	0.188	[−0.037; 0.414]
PSYRATS voices scale (Unblinded)	93	1.052	1.688	0.533	0.079	[−0.169; 0.327]
*Target mechanisms*						
ERQ expressive suppression (SR)	88	2.739	1.213	0.024 *	0.501	[0.066; 0.935]
ERQ reappraisal (SR)	87	5.379	1.817	0.003 **	0.723	[0.244; 1.202]
ERSQ total score (SR)	88	−4.378	3.407	0.199	−0.248	[−0.626; 0.130]
ERI positive emotions (SR)	89	−3.019	1.788	0.091	−0.299	[−0.646; 0.048]
ERI negative emotions (SR)	89	1.260	2.426	0.603	0.115	[−0.319; 0.550]
PSWQ sum score (SR)	89	−10.942	3.132	<0.0001 **	−0.869	[−1.356; −0.381]
ISI insomnia sum score (SR)	87	−3.156	1.063	0.003 **	−0.506	[−0.839; -0.172]
RSES sum score (SR)	89	1.533	1.049	0.144	0.222	[−0.076; 0.519]
SCS total score (SR)	88	4.415	2.797	0.114	0.279	[−0.067; 0.625]
BCSS negative-self (SR)	88	0.473	0.890	0.595	0.083	[−0.224; 0.391]
BCSS positive-self (SR)	88	−0.836	1.034	0.419	−0.156	[−0.533; 0.222]
BCSSS negative-others (SR)	87	−1.034	1.115	0.354	−0.179	[−0.557; 0.199]
BCSS positive-others (SR)	88	0.522	1.086	0.630	0.120	[−0.370; 0.610]

Regression analysis of the post-scores (dependent variable) on the group (independent variable: 0 = WL, 1 = CBTd-E) while controlling for study site.

*SE* standard error, *SR* self-rating, *PSYRATS* psychotic rating symptom scales, *PDI* Peters et al. delusions inventory, *EMA* ecological momentary assessment of persecutory delusions over 6 consecutive days and 10 time points: mean score, *CPZ* chlorpromazine equivalent of antipsychotic medication, *ERQ* emotion regulation questionnaire, *ERSQ* emotion regulation style questionnaire, *ERI* emotion regulation inventory, *PSWQ* Penn State worry questionnaire, *ISI* insomnia severity index, *RSE* Rosenberg self-esteem scale, *SCS* self-compassion scale, *BCSS* brief core schema scale.

^a^Unstandardized beta.

*p < 0.05; **p < 0.01.

Results from the regression-based ANCOVA using FIML for addressing missing data at 6-months post-assessment (T3) are presented in Table 4. No statistically significant effects favoring CBTd-E were found concerning the primary outcome, delusions. Nevertheless, descriptive effect sizes indicate medium effects (PSYRATs delusions sum scale: *d*_blind_ = −0.45; *d*_unblinded_ = −0.64).

**Table 4:** Results of the regression-based ANCOVA with Full Information Maximum Likelihood (FIML) for missing data handling at 6-months post-assessment (T3) on the primary and secondary outcome variables in the intent-to-treat sample (ITT: *n* = 94).

Measure	*n (analysis)*	Group differences *beta*_unst_.^a^ *SE*		*p*	Effect size *d 95% CI*	
*Primary outcome variables*						
PSYRATS delusions scale (Blind)	61	−2.145	1.955	0.273	−0.451	[−1.258; 0.355]
PSYRATS delusions scale (Unblinded)	93	−2.236	1.435	0.119	−0.635	[−1.435; 0.164]
*Secondary outcome variables*						
PDI-21 grand total score (SR)	88	−8.548	9.123	0.349	−0.150	[−0.464; 0.164]
EMA persecutory delusions (SR)	72	−0.154	0.239	0.518	−0.110	[−0.446; 0.225]
PANSS POS (Blind)	62	−1.527	1.004	0.128	−0.341	[−0.779; 0.098]
PANSS POS (Unblinded)	93	−1.760	0.840	0.036 *	−0.468	[−0.910; −0.030]
PANSS NEG (Blind)	62	0.034	1.273	0.979	0.007	[−0.541; 0.556]
PANSS NEG (Unblinded)	93	−0.536	0.977	0.583	−0.112	[−0.514; 0.289]
PANSS GEN (Blind)	62	−3.755	1.806	0.038*	−0.558	[−1.084; −0.032]
PANSS GEN (Unblinded)	93	−3.018	1.535	0.049*	−0.468	[−0.935; −0.002]
CDSS total score (Blind)	62	−1.074	1.077	0.319	−0.291	[−0.864; 0.281]
CDSS total score (Unblinded)	93	−2.301	0.702	0.001 **	−0.540	[−0.862; -0.217]
RFS general functioning (Blind)	61	0.321	0.589	0.586	0.147	[−0.381; 0.675]
RFS general functioning (Unblinded)	93	1.048	0.506	0.038*	0.411	[0.022; 0.801]
RFS social functioning (Blind)	61	0.855	0.590	0.147	0.391	[−0.137; 0.920]
RFS social functioning (Unblinded)	93	0.868	0.536	0.105	0.372	[−0.078; 0.822]
CPZ	93	−123.283	165.160	0.455	−0.245	[−0.890; 0.399]
PSYRATS voices scale (Blind)	61	−2.895	2.935	0.324	−0.244	[−0.727; 0.240]
PSYRATS voices scale (Unblinded)	93	−1.822	2.037	0.371	−0.137	[−0.436; 0.163]
*Target mechanisms*						
ERQ expressive suppression (SR)	88	1.198	1.060	0.258	0.219	[−0.161; 0.599]
ERQ reappraisal (SR)	87	4.371	1.391	0.002 **	0.588	[0.221; 0.954]
ERSQ total score (SR)	88	0.282	3.758	0.940	0.016	[−0.401; 0.433]
ERI positive emotions (SR)	89	−3.557	1.897	0.061	−0.352	[−0.720; 0.016]
ERI negative emotions (SR)	89	−2.541	2.776	0.360	−0.232	[−0.730; 0.265]
PSWQ sum score (SR)	89	−6.556	3.347	0.050 *	−0.520	[−1.041; 0.000]
ISI insomnia sum score (SR)	87	−3.074	1.250	0.014 *	−0.493	[−0.885; −0.100]
RSES sum score (SR)	89	2.517	1.163	0.030 *	0.364	[0.034; 0.693]
SCS total score (SR)	88	1.162	2.895	0.688	0.073	[−0.285; 0.432]
BCSS negative-self (SR)	88	−0.073	1.025	0.944	-0.013	[−0.367; 0.342]
BCSS positive-self (SR)	88	0.054	1.165	0.963	0.010	[−0.415; 0.436]
BCSSS negative-others (SR)	87	−1.297	1.112	0.243	−0.224	[−0.601; 0.153]
BCSS positive-others (SR)	88	−0.049	1.158	0.966	−0.011	[−0.534; 0.511]

Regression analysis of the post-scores (dependent variable) on the group (independent variable: 0 = WL, 1 = CBTd-E) while controlling for study site.

*SE* standard error, *SR* self-rating, *PSYRATS* psychotic rating symptom scales, *PDI* Peters et al. delusions inventory, *EMA* ecological momentary assessment of persecutory delusions over 6 consecutive days and 10 time points: mean score, *PANSS* positive and negative syndrome scale, *PANSS POS* PANSS positive scale, *PANSS NEG* PANSS negative scale, *PANSS GEN* PANSS general psychopathology scale, *CDSS* Calgary depression rating scale, *RFS* role functioning scale, *CPZ* chlorpromazine equivalent of antipsychotic medication, *ERQ* emotion regulation questionnaire, *ERSQ* emotion regulation style questionnaire, *ERI* emotion regulation inventory, *PSWQ* Penn State worry questionnaire, *ISI* insomnia severity index, *RSE* Rosenberg self-esteem scale, *SCS* self-compassion scale, *BCSS* brief core schema scale.

^a^Unstandardized beta.

*p ≤ 0.05; **p ≤ 0.01.

The blinded assessment of secondary outcome variables revealed a statistically significant effect on general psychopathology (PANSS GEN: *d*_blind_ = −0.56), but not on any other secondary outcome measures. In the unblinded assessment, significant effects were noted on positive symptoms (PANSS POS: *d*_unblind_ = −0.47), general psychopathology (PANSS GEN: *d*_unblind_ = −0.47), depression (CDSS total score: *d*_unblind_ = −0.54), and general functioning (RFS general functioning: *d*_unblind_ = 0.41). However, no statistically significant effect was found on negative symptoms (PANSS NEG), social functioning (RFS social functioning), antipsychotic dosage (chlorpromazine equivalent), or hallucinations (PSYRATS voices scale).

### Change in target mechanisms

Regarding change in the target mechanisms at T2 (Table 3), there were significant effects favoring CBTd-E in expressive suppression (*d* = 0.50), cognitive reappraisal (*d* = 0.72), worry (PSWQ sum score: *d* = −0.87), and quality of sleep (ISI sum score: *d* = −0.51).

Regarding change in the target mechanisms at T3 (Table 4), we found significant effects in favor of the CBTd-E group in cognitive reappraisal (ERQ reappraisal: *d* = 0.59), worry (PSWQ sum score: *d* = −0.52), quality of sleep (ISI sum score: *d* = −0.49), and self-esteem (RSE sum score: *d* = 0.36). Other measures of affect regulation and maladaptive schemata did not improve significantly, but descriptive effect sizes suggest small to medium effects in the expected direction.

### Sensitivity analysis

At T2, the results of linear regression analysis with single imputation (Table S4) and listwise deletion (Table S5) as missing value replacement strategies mirrored the results of the FIML analysis.

The results of the linear regression analysis employing single imputation as a missing data strategy at T3 (Table S6) largely mirrored those of the FIML analysis, except that CBTd-E showed no statistically significant effect on RFS general functioning (unblinded) or the PSWQ sum score, but had a significant effect on the ERI positive emotions score. The results of the linear regression analyses using listwise deletion at T3 (Table S7) also largely mirrored the findings of the FIML analysis. However, no statistically significant effect was observed on PANSS GEN (blinded and unblinded), RFS general functioning (unblinded), or the PSWQ sum score.

### Additional exploratory analysis of post-intervention effects

Additional exploratory regression analyses were conducted, incorporating the six subscales of the PSYRATS delusions scale (blinded assessment) at T2 (see Table S8 for the means and standard deviations of the PSYRATS subscales). At T3 (Table S9), the FIML analysis indicated that patients who received CBTd-E reported a significantly reduced preoccupation with delusions. However, they did not show improvement in the other subscales. Single imputation (Table S10) and listwise deletion (Table S11) analyses mirrored these findings.

Additionally, to assess whether baseline scores in affect regulation and negative self-schemata moderate the effect of the intervention on delusions at post-assessment (T3) while controlling for baseline delusion scores and center effects, we conducted two exploratory moderation analyses by extending the main regression model by baseline scores of affect regulation scores (ERI negative emotions score, model 1) and baseline scores in negative self-schemata (BCSS negative self-schemata, model 2).

The ERI negative emotions score was used as a global measure for affect regulation, as it includes various functional and dysfunctional emotion regulation strategies for negative emotions^48^. The BCSS negative self-score was used because it was specifically designed to measure core negative self-schemata^49^. A significant interaction term would indicate that the intervention’s effect on delusions varied depending on patients’ initial affect regulation levels or self-schemata. Neither of the moderation analyses at T3 showed a statistically significant moderation effect (model 1: see Table S12, Table S13, and Table S14, model 2: see Table S15, Table S16, and Table S17).

### Manual adherence and therapists’ competency

In the CBTd-E completer group (*n* = 34; see Fig. 1), patients received an average of 4.97 diagnostic assessment and preparation sessions (*SD* = 1.09; range: 2–6) and 17.35 sessions of CBTd-E (*SD* = 5.89; range: 5–25). Therapists allocated an average of 11.44 sessions to the *affect regulation module* (*SD* = 4.48) and 5.65 sessions to the *maladaptive schemata* module (*SD* = 3.65). They reported deviating from the manual in 0.50 sessions (*SD* = 0.79; range: 0–3 sessions), with the most common reason for deviation being the need to address an acute crisis or adverse event (59%). The 13 patients who discontinued CBTd-E received an average of 4.08 diagnostic assessment and preparation sessions (*SD* = 1.12; range: 3–6) and 4.38 sessions of CBTd-E (*SD* = 5.21; range: 0–12).

Table S18 shows the mean scores and standard deviations for the CTS-RP scales derived from 110 randomly selected audio recordings. The mean scores varied from 4.1 to 4.8 across different domains of competence, indicating moderate to high levels of therapist competency.

## Discussion

Aiming to reach stronger effects on delusions than the small effects achieved in standard CBTp, we tested the efficacy of CBTd-E, an emotion-oriented version of CBT, with one module focusing on affect awareness, regulation, and stability, and the other module focusing on maladaptive schemata.

The primary analysis, however, did not indicate a benefit of CBTd-E over the waitlist group in reducing the PSYRATS delusions subscale score. The magnitude of the descriptive effect size on the PSYRATS delusions subscale was small to medium (*d* = −0.45), similar to the effect observed in the six-week worry intervention^8^ (*d* = −0.47) that also targeted the affective pathway, but focused on worry only. The effect is less pronounced than the effect on the changes in delusions achieved by the ‘Feeling Safe’ program^50^, which was pre-registered after we had planned and received funding for the present study. The ‘Feeling Safe’ program^50^ combined modularized interventions focusing on both the cognitive and affective pathway to delusions and placed a strong emphasis on behavioral interventions to change safety behaviors. It involved six modules aiming at improving reasoning, feeling safe, quality of sleep, worry, voices, and self-confidence over six months. In this program, three to four modules were selected based on patients’ distress and preferences. The ‘Feeling Safe’ program showed large effects on delusions, both in the primary outcome, delusion conviction (*d* = −0.86), and on the PSYRATS delusions scale (*d* = 1.20) in comparison to an active control group (befriending). The magnitudes of the effect sizes in the more distal outcomes, such as other symptoms, were similar to those found in our study. However, the ‘Feeling Safe’ study differed from our approach not only in regard to the targets and types of interventions, but also in relation to design aspects, rendering a direct comparison of effects difficult. For example, our study did not limit inclusion to persecutory delusions, but included patients with different types of delusions. Also, we did not restrict participation to patients with a minimum level of delusion conviction, but required delusions to manifest with mild to moderate severity in several delusion dimensions, which did not necessarily have to include the conviction dimension. These sample differences may have accounted for the lower effect size in our study. However, they are unlikely to be the main factor, as 75.53% of the patients we included reported persecutory delusions and 69.15% reported a PSYRATS delusions conviction score of at least 3 (i.e., high conviction of 50–99%, see Table 1) which corresponds to the criterion used in the ‘Feeling Safe’ program (at least 60% conviction) and to some of the targeted studies preceding it^7,8^.

Regarding secondary outcome variables, CBTd-E produced a significant effect on general psychopathology. The effects on other secondary outcomes were either non-significant or only significant in the unblinded assessments (positive symptoms, depressive symptoms, general functioning). This pattern of outcomes remained consistent in additional sensitivity analyses and aligns with our pilot study, where we also found improved general psychopathology^47^.

In contrast to the pilot study^47^, this modified version of CBTd-E was more effective in addressing the target mechanisms of change: affect regulation and maladaptive schemata. Participants who received CBTd-E showed a significantly stronger increase in the use of adaptive emotion regulation skills (*d* = 0.59), a notable reduction in worry and rumination (*d* = −0.52), improved quality of sleep (*d* = −0.49), and enhanced self-esteem (*d* = 0.36) at 6-month post-assessment. These effects are comparable to those of the ‘targeted’ studies (worry: *d* = −0.47^8^; self-esteem: *d* = 0.62^7^).

Furthermore, the significant effects on emotion regulation, worry, and quality of sleep were already evident by the 3-month post-assessment (T2) following the administration of the module designed to enhance affect regulation. In contrast, self-esteem improved only after the 6-month post-assessment, after the reception of the second module focused on maladaptive schemata. Consequently, the proposed intervention targets improved outcomes following administration of the corresponding modules.

Although the intervention did not significantly reduce delusions, the fact that CBTd-E improved its targeted areas—affect regulation and maladaptive self-schemata—is, in our view, meaningful in its own right. This holds true, in particular, for the increases in self-esteem and self-worth, since traditional CBTp interventions show inconsistent effects on global self-esteem^51–53^ and ‘targeted’ CBT modules with a focus on schemata have not succeeded in maintaining achieved changes in self-esteem/self-schemata at follow-ups^7,50^.

Better emotion regulation strategies, reduced worry, improved sleep quality, and a more positive self-image could improve patients’ well-being and quality of life, even if they do not affect delusional conviction and distress. The relevance of these factors in their own right is supported by the fact that patients have emphasized affective factors^21,54^ and improved coping with distressing emotions^20,21,55^ as more important intervention goals in comparison to symptom reduction. In addition, better affect regulation and less maladaptive schemata and an improved quality of life and well-being may improve delusional distress and conviction over a longer time period than the time period we measured in the present study. In conclusion, CBTd-E could serve as a valuable additional intervention option for patients with delusions who prioritize emotional well-being, enhanced emotion regulation, and self-esteem.

### Limitations

A limitation is that we did not quite reach our recruiting target of 102 patients, which diminished the study’s statistical power. While having greater power may have rendered the effect size for the primary outcome (*d* = 0.45) statistically significant, the achieved effect still falls short of the expected boundary for at least a moderate effect. The extensive baseline assessment, including video recordings of the assessment and intervention, might have introduced recruitment bias, leading to fewer patients with severe delusions enrolling, potentially impacting the sample’s representativeness. However, video recording was not mandatory for participation, and some patients with delusions took part in the trial and intervention without undergoing video assessment. Relatedly, the loosening of the in-/exclusion criteria at the beginning of the trial should be noted, which broadened the range of included delusion severity and may have thereby also reduced the power to detect an effect on the primary outcome.

Another limitation is the high number of missing data for the observer-rated measures. This issue was partly due to a fairly high drop-out rate in the CBTd-E group of 27%. However, almost half of the drop-outs were participants who withdrew shortly after the baseline assessment and during the detailed assessment required to obtain coverage of the therapy within the German healthcare system. To our knowledge, this procedure is unique to the German healthcare system and is not necessary in fully funded trials, where drop-out rates typically refer to those who actually initiated therapy. Another reason for the high number of missing data was technical problems and the absence of blinded measures in the third center. This center had volunteered to participate without funding, and the inability to provide participant remuneration for time spent on assessment, along with a lack of technical support, resulted in a breakdown of the blinding procedure at that center. We addressed these issues using FIML^56,57^, by reporting the unblinded findings and conducting additional sensitivity analyses, which do not indicate that the missing data significantly biased the overall pattern of findings.

In addition, several outcome variables were assessed only at baseline (T1) and post-assessment (T3), precluding the use of mixed-effects ANCOVA models for all outcomes. Although such models could have been applied to outcomes with three assessments, this would have resulted in heterogeneous estimands across outcome domains. To ensure consistency and comparability of treatment effect estimates, we therefore applied a uniform baseline-adjusted ANCOVA framework.

Additionally, our trial therapists were still undergoing their postgraduate CBT training. Due to fluctuations in study centers, many therapists treated only 1–5 patients, providing limited opportunities for skill development over time, and this might have limited the observed treatment effect, as more experienced therapists might be more adept at delivering the intervention^58^. In contrast, most other ‘targeted’ studies involved a small number of more specialized therapists (‘Feeling Safe’: 9 therapists for 130 patients^50^; ‘Slow Mo’: 11 therapists for 181 patients)^17^, which likely increased therapist expertise and efficacy, but also presents a challenge in maintaining effects during clinical implementation.

Given the limited funding, we also did not involve individuals with lived experience in the trial, which could have improved the feasibility of the assessments and the intervention. Finally, we did not incorporate a follow-up assessment and cannot confirm whether our observed effects are stable over longer time periods than the 6 months covered by the intervention.

### Implications of the study results

In light of our results, it can be questioned whether our approach of focusing on two affective pathways (affect regulation and maladaptive schemata) in order to impact delusions is worth further pursuing. Improving the therapy manual in light of insights derived from the pilot study is likely to have produced the stronger effects on affect regulation and maladaptive schemata compared to the pilot study. Changes in these outcomes are promising per se, even in the absence of symptom improvement. However, more pronounced and broader effects on these targets may be necessary to achieve the changes in delusions we aimed for. One way to intensify the intervention could be to provide patients with supplementary online material and training opportunities aligned with the CBTd-E interventions. This would help them recapitulate the sessions’ content and support the home practice of new techniques. This support could be provided by a web application on patients’ smartphones, similar to the ‘Slow Mo’-RCT^17^.

Another option is to enhance the alignment of the intervention with the patient’s profile concerning the potential intervention targets. While there was some element of personalization in our study through individual selection of interventions within the modules, the modules themselves, along with their sequence and duration, were fixed (3 months for each module), and patients were not pre-selected based on specific problems in the target areas.

To be suitable for a broader spectrum of patients with delusions, modularized approaches must ensure they have an adequate number and appropriate types of modules to provide a good fit for each patient. Thus, it could be promising to combine the CBTd-E modules with modules from other targeted interventions (i.e., reasoning modules) and assess in RCTs whether offering a wider range of target modules for patients to select from based on their personal needs and problems, or informed by a personalized treatment formulation of their delusions, proves beneficial.

### Summary and conclusions

CBTd-E did not show a statistically significant effect on delusions and can therefore not be recommended as an evidence-based specific intervention for delusions. Nevertheless, it led to notable improvements in general psychopathology. It also improved affect regulation and maladaptive schemata and could thus be suitable for patients aiming for improvements in these domains. Future work could test the effects of incorporating the modules into a larger intervention program using a personalized module selection to better align CBTd-E with patients’ problem profiles.

## Methods

### Ethical approval and registration

The ethics commission of the German Society of Psychology (DGPs) approved the study (Approval TL012015 & TL022016). The study was registered at ClinicalTrials.gov (Identifier NCT02787135; https://clinicaltrials.gov/study/NCT02787135, Date of registration: 25th of May 2016) and funded by the German Research Foundation (DFG). The registration included (1) baseline analyses comparing non-clinical controls and patients that have been published elsewhere (emotion regulation: ^25,27,59^; sleep: ^35^); (2) the main trial outcomes, which we report on fully here; (3) mediation analysis, of which we report the post-treatment effects on the main target mechanisms. The full mediation analysis, using all time points, will be disseminated elsewhere.

### Study design

The study design was a single-blind, randomized controlled parallel group trial conducted at three study centers (Bochum, Marburg, Hamburg) comparing individual CBTd-E plus standard care (CBTd-E group) with patients on a waitlist receiving standard care (WL group). All outcome variables were assessed at baseline (T1). At three months post-assessment (T2), patients were assessed with the *Psychotic Symptom Rating Scale* (PSYRATS)^60^, Ecological Momentary Assessment (EMA), and completed self-rating questionnaires. All outcome variables were assessed again at six months post-assessment (T3). There was no patient or public involvement in the trial design.

### Participants

Participants were recruited at the outpatient clinics of three university psychology departments in Hamburg, Marburg, and Bochum. The inclusion criteria were:

1. Age between 16 and 70 years;
2. Diagnosis of schizophrenia, schizophreniform disorder, schizoaffective disorder, delusional disorder, brief psychotic disorder, and schizotypal personality disorder as assessed with the Structured Clinical Interview for Mental Disorders (SCID-5-CV)^61^;
3. Delusions had to be present as indicated by (1) a score ≥2 in at least 3 of the 6 items of the delusions subscale of the PSYRATS^60^ (related to the last week); (2) confirmed by the SCID-5-CV^61^ (for the last 3 months). The cut-off score on the PSYRATS was selected to ensure that we include patients with delusions that manifest to at least a mild to moderate degree on several dimensions (amount of distress, distress intensity, conviction, amount of preoccupation, preoccupation intensity, and disruption);
4. Fluency in the German language;
5. No acute suicidal ideation;
6. No comorbid diagnosis of a substance use disorder in the last 6 months, as this would require a transfer to a specialized patient unit in the German healthcare system;
7. No comorbid borderline personality disorder or Benzodiazepine use, as these were expected to significantly interfere with the emotion-oriented intervention approach.

### Recruitment procedure and diagnostic assessment

Recruitment began in July 2016 and ended in September 2018. Unblinded assessments were completed at the post-assessment (T3) in September 2019. Blinded assessments were performed on the recorded videos of the unblinded assessments and were completed in December 2021. Patients contacted the outpatient clinic directly or were referred by their psychiatrist, general practitioner, or social worker, who had been informed about the therapy trial. Candidate patients met with a clinical psychologist from the research team (LD, LL, TT). They were informed about the study, its duration, and the focus of the interventions on affect regulation and maladaptive schemata. Following written informed consent, patients were assessed for eligibility using the Positive and Negative Syndrome Scale (PANSS)^62^ and the PSYRATS^60^ interview. After randomization, all other diagnostic assessments were conducted by study assistants. Assessments also included a detailed diagnostic assessment required for the German health insurance to cover the costs and additional procedures in Marburg and Hamburg that included behavioral paradigms, psychophysiological assessments, EMA, and actigraphy assessments, of which baseline comparisons have been reported elsewhere^25,27,35,59^.

### Randomization procedure

Randomization to the CBTd-E or the WL group (1:1) was conducted utilizing a permuted block algorithm with a fixed block size of four numbers, stratified by study center and symptom severity, based on the PANSS^63^ total score (mild [0–53 points], moderate [54–74], or severe [>74]). Random numbers were generated via a website (www.random.org) and recorded in a table prior to the commencement of the study. Participants were assigned randomly by an independent rater who received an email from the study assistant containing the patients’ PANSS total scores.

### Emotion-oriented cognitive behavior therapy for delusions (CBTd-E)

CBTd-E was offered in approximately 25 weekly individual 50-min sessions over 6 months and consisted of two modules: *affect regulation* and *maladaptive schemata*.

Therapists aimed to establish a collaborative partnership grounded in mutual respect. This was done through an open, transparent, person-centered, and genuinely empathic approach, in which therapists proactively addressed mistrust, were open to providing information about themselves, and acknowledged the patient’s perspective as understandable given their experiences. In addition, therapists and patients engaged in collaborative goal-setting and in guided discovery of strategies that patients might find helpful.

The first module, *affect regulation*, included: (1) psychoeducation on emotions (i.e., how to identify and label emotions correctly and how to become aware of the related thoughts, perceptions, and body sensations) and implementing an emotion diary, (2) psychoeducation on functional and dysfunctional ER strategies, (3) challenging maladaptive beliefs about emotions, (4) training functional ER strategies for specific emotions (e.g., improving awareness, acceptance, reappraisal) and reducing worrying (i.e., by challenging positive and negative metacognitions on worrying, replacing worrying with alternative coping strategies), and (5) encouraging patients to adjust their daily schedule to improve their emotional resilience (which involved psychoeducation on mood, the importance of a regular daily schedule and implementation of a mastery and pleasure diary, the implementation of a regular sleep schedule, a relaxing bedtime routine, and a sleep-improving environment). The specific interventions for this module were selected from manuals on CBT, Acceptance and Commitment Therapy, Emotion-Focused Therapy, and Metacognitive Therapy^64–68^.

The second module, *maladaptive schemata*, included (1) psychoeducation on self-esteem and self-acceptance and the role of positive and negative self-schemata along with implementing a diary to monitor self-schemata and related cognitions, emotions, and behavior, (2) challenging negative self-schemata and related cognitions and behavior using Socratic questioning, chair work, imagination strategies, behavior experiments, and helpful self-instructions. In the final session of CBTd-E, interventions and helpful coping strategies were reviewed. Interventions for this module were selected from Person-based Cognitive Therapy and Compassion-Focused Therapy^69,70^.

Patients received the *affect regulation* module during the first three months and the *maladaptive schemata* module in the following three months. Therapists were instructed to adhere closely to the manual. However, the therapists were free to adjust the manual’s interventions within each module to the needs or symptom profile of their respective patients.

*Standard care* in Germany usually involves regular monthly visits to a psychiatrist, where patients discuss their mental health and medication-related issues.

### Training of therapists

Therapists were 24 clinical psychologists (M.Sc. or diploma) enrolled in their second to third year of German postgraduate training to become certified CBT therapists (total duration: 3–5 years). All therapists had received 200–300 h of theoretical postgraduate CBT training and had passed an examination that qualified them to treat outpatients under the supervision of a certified CBT therapist. All therapists received an additional 20 h of training in the CBTd-E approach, consisting of lectures and role-plays supervised by S.M. and biweekly manual supervision by S.M. After the training, therapists had ongoing access to an online training platform that provided all training lectures, such as screencasts and additional written material.

## Measures

### Assessment, training, and blinding procedure

Unblinded assessments were conducted and recorded on video by trained raters. After the conclusion of the study, L.L. randomized these recordings, and the blinded raters rated them. To ensure objectivity and maintain blind ratings, raters had no prior contact with the patients, and the assessment recordings for each patient were distributed randomly among independent raters. L.L. later unblinded the ratings. All raters completed at least 10 h of training, including supervision and the assessment of two training patients.

### Outcome measures

The reliability of the observer-rated scales was reported as intraclass correlations (ICC) between ratings of scientific assistants and blinded raters in the sample at baseline assessment (T1) (*n* between 60 and 62 patients). It is calculated using an unadjusted analysis of variance. The reliability of self-rated instruments is reported as internal consistency (Cronbach’s alpha) in the sample at T1.

### Primary outcome

The delusions scale of the *Psychotic Symptom Rating Scale (PSYRATS)*^60^ (range: 0–24) was utilized to evaluate the primary outcome delusions. Inter-rater reliability and convergent validity have been reported as excellent in patients with psychotic disorders^60^. Baseline intraclass correlations in our sample were acceptable *(ICC* = 0.85).

### Secondary outcome measures

Self-rated delusional beliefs were assessed with the *Peters* et al. *Delusions Inventory – short version (PDI-21)*^71^. The *PDI-21 grand total score* was used, which is the sum score of endorsed beliefs, preoccupation, distress, and conviction (range: 0–336). Internal consistency and both convergent and discriminant validity were reported as excellent in a sample of non-clinical controls and inpatients with psychotic disorders^71^. Internal consistency was also excellent in our sample (Cronbach’s alpha = 0.96).

The *Ecological Momentary Assessment (EMA) of paranoid delusions* was used to assess patients’ state paranoia in daily life based on six items that were answered at 10 random times a day over six days. The EMA paranoia mean score was used (range 1–7). Internal consistency was acceptable in our sample (Cronbach’s alpha = 0.79) for the within-subject level and excellent (0.96) for the between-subject level. More information on the EMA items can be found on Table S1.

*The PANSS*^62^ interview was used to assess observer-rated positive symptoms, negative symptoms, and general psychopathology of schizophrenia. We report the *PANSS positive scale* (*PANSS POS:* 7 symptoms, range: 7–49), the *PANSS negative scale* (PANSS NEG: 7 symptoms, range: 7–49), and the *PANSS general psychopathology scale* (PANSS GEN: 16 symptoms, range: 16–112). In a recent meta-analysis based on 119 publications, internal consistency and inter-rater reliability were found acceptable^72^. Baseline intraclass correlations in our sample were acceptable (PANSS positive scale: *ICC* = 0.81; PANSS negative scale: *ICC* = 0.72; PANSS general psychopathology: *ICC* = 0.69).

The sum score of the *Calgary Depression Rating Scale for Schizophrenia (CDSS*, German version^73^*)* was used to assess observer-rated depressive symptoms (range 0–33). Baseline intraclass correlations were adequate to excellent in three validation studies^73^ and good in our sample (*ICC* = 0.89).

*The Role Functioning Scale (RFS)*^74^ was used to assess observer-rated functioning in the areas of working productivity, independent living, immediate social network relationships (friends and family), and extended social network relationships (other social contacts). The mean scores are reported for *general functioning* and *social functioning* (range: 0–12). Inter-rater reliability was acceptable, and the scale was found to discriminate well between patients with psychotic or depressive disorders and non-clinical controls^74^. The baseline intraclass correlation in our sample was good for both scales (RFS general functioning: *ICC* = 0.82; RFS social functioning: *ICC* = 0.81).

*Antipsychotic medication dosage* per day was assessed and computed as Chlorpromazine equivalent^75^.

The *PSYRATS voices subscale sum score*^60^ was used to assess observer-rated auditory hallucinations (PSYRATS voices scale: range: 0–44). Inter-rater reliability and convergent validity have been reported as excellent in patients with psychotic disorders^60^. Baseline intraclass correlations in our sample were excellent (*ICC* = 0.98).

### Target mechanisms of change

*Affect regulation*. To assess different aspects of emotion regulation, we used the 10-item *Emotion Regulation Questionnaire* (ERQ, German version^76^) that is subdivided into the subscales *ERQ expressive suppression* (sum score of 4 items: range: 0–24) and *ERQ cognitive reappraisal* (sum score of 6 items: range: 0-36). Convergent validity was good and internal consistency was acceptable in a student sample^76^. In our sample, we also found acceptable internal consistency (ERQ expressive suppression: Cronbach’s alpha = 0.73; ERQ cognitive reappraisal: 0.80).

In addition, we used the *ERSQ* sum score of the *Emotion Regulation Skills Questionnaire (ERSQ*, German version^77^*)* that assesses emotion regulation skills in nine dimensions (awareness, clarity of emotions, physical sensations, understanding, acceptance, resiliency, self-support, readiness to confront emotions, and emotion regulation) using 27 items answered on 5-point Likert scales (range: 0–108). Construct validity and internal consistency were satisfying to excellent in different samples^77^. Internal consistency of the ERSQ total score was excellent in our sample (Cronbach’s alpha = 0.92).

Further, we used the *Emotion Regulation Inventory* (ERI, German version^48^) that includes 38 items using 5-point Likert scales that are subdivided into two subscales that subsume strategies used to regulate positive (*ERI positive emotions*, 16 items; range: 0–64) and negative emotions (*ERI negative emotions*, 22 items; range: 0–88). Convergent and construct validity and internal consistency were satisfying for both subscales in a student sample^48^. Internal consistency was good for both subscales in our sample (ERI positive emotions: Cronbach’s alpha = 0.82; ERI negative emotions: 0.75).

The total score of the *Penn State Worry Questionnaire* (PSWQ German version^78^) was used to measure excessive worrying and rumination with 15 items rated on 7-point Likert scales (range: 0–90). Construct validity was acceptable, and internal consistency was excellent in a student sample^78^. Internal consistency was good in our sample (Cronbach’s alpha = 0.86).

Finally, we used the *Insomnia Severity Index* (ISI)^79^ to assess sleep problems. The ISI has seven items answered on 5-point Likert scales (range: 0–28). Construct validity was acceptable, and internal consistency was excellent for the German version in non-clinical controls^80^. Internal consistency was good in our sample (Cronbach’s alpha = 0.85).

*Maladaptive schemata*. We used the sum score of the *Rosenberg Self-Esteem Scale* (RSES, German version^81^) to assess global self-esteem. The RSE consists of 10 items that are rated on 4-point Likert scales, resulting in a sum score (range: 0–30). Construct validity was good, and internal consistency was excellent in non-clinical controls^81^. In our sample, internal consistency was also good (Cronbach’s alpha = 0.90)

We used the *Self-Compassion Scale* sum score *(SCS*, German version^82^) to assess self-compassion and self-kindness. The SCS has 26 items answered on 5-point Likert scales (range: 0–104). Construct validity and internal consistency were good in a student sample^82^. Internal consistency was excellent in our sample (Cronbach’s alpha = .90).

The *Brief Core Schema Scale* (BCSS)^49^ consists of 24 items assessing positive and negative self-schemata and positive and negative other-schemata (6 items each). Items are answered on 5-point-Likert scales. The four subscales *BCSS negative-self* (negative self-schemata), *BCSS positive-self* (positive self-schemata), *BCSS negative-others* (subsuming negative schemata on other persons), and *BCSS positive-others* (positive schemata on other persons) were used (each range 0–24). Construct validity and internal consistency were good in non-clinical controls and patients with psychotic disorders^49^. In our sample, internal consistency was good (BCSS negative-self: Cronbach’s alpha = 0.86; BCSS positive-self: 0.86; BCSS negative-other: 0.90; BCSS positive-other: 0.86).

Serious adverse events were defined as symptom deterioration and hospital readmission and were assessed systematically by individual therapists.

### Assessment of manual adherence and therapeutic competency

After every session, therapists completed a brief protocol on the manualized CBTd-E interventions they used and were asked to report any deviation from the manual and adverse events. In addition, all sessions were audiotaped, provided patient consent (which was not mandatory for trial participation), and 1 in 5 were randomly selected and assessed by five trained raters using an adapted version of the unpublished revised version (*Cognitive Therapy Scale-Revised for Psychosis (CTS-RP))* that was based on the Cognitive Therapy Scale for Psychosis (CTS-Psy)^83^. CTS-RP raters received ten hours of training on the CTS-RP manual and the study manual. The CTS-RP included 13 items rated on 7-point Likert scales (range 0–6). They assessed the therapists’ competency regarding agenda setting, feedback, collaboration, effective time use, positive focus, interpersonal effectiveness, assessing key emotions, cognitions, and behavior, guided discovery, use of CBTd-E strategies, homework, and estimated general competency. Inter-reliability in the as-treated sample based on 13 audios that were rated by 2 raters was acceptable (*ICC* = 0.77), and internal consistency was also acceptable (Cronbach’s alpha = 0.76).

### Sample size considerations and power analysis

As the present study focused on the affective pathway to delusions and aimed to reach stronger effects on delusions than the small effects achieved in standard CBTp^5^, our intervention aimed for an at least moderate effect size on delusions in line with the mean effect sizes for the ‘targeted’ studies we found in our meta-analysis (*d* = 0.51)^4,5^ and their low drop-out rate of 10%^7,8^. A sample size calculation for an ANCOVA was computed using a formula recommended by Borm^84^ and the program G-power^85^. Based on a computed adequate sample size for a two-sided *t*-test for independent groups (alpha error: 0.05, test power: 0.80), a recommended sample size for an ANCOVA would be 93 patients (using the recommended formula and assuming a correlation between pre- and post-scores of *r* = 0.66 as derived from the pilot study). Taking drop-out into account, we aimed for 102 patients.

### Changes to the protocol as registered

Since recruiting patients with delusions proved more challenging than anticipated, we were only able to recruit 94 patients during the funding period. At one center (Bochum), the blinded video assessments could not be conducted for technical reasons and/or because patients refused the video recordings. As a result, blinded assessment data and EMA data were missing for that center. Therefore, we will report the effects based on the blinded and unblinded assessments in the tables.

Also, we had initially planned to include only patients with (1) at least a moderate score (≥3) in 3 out of 6 items on the PSYRATS^60^ delusions subscale, (2) above a minimum score on scales assessing the target mechanisms, (3) a diagnosis of schizophrenia, schizoaffective disorder and delusional disorder and (4) an estimated general intelligence score of at least 70. However, when the first wave of recruitments within the first 2 months indicated unexpectedly high rates of exclusion that threatened the feasibility of the trial, we decided to slightly loosen the inclusion criteria to (1) include patients with at least a mild score (≥2) in 3 out of 6 items on the PSYRATS^60^ delusions subscale, (2) not to exclude patients scoring below predefined thresholds on scales assessing the target mechanisms, (3) to also include patients with brief psychotic disorders or schizotypal disorders, and (4) not to exclude those with an estimated general intelligence score below 70.

Finally, in addition to the pre-registered design, the waitlist (WL) group received the CBTd-E intervention following a six-month waiting period, with additional evaluations conducted at nine months (T4) and twelve months (T5). This was done to enable the analysis of exploratory pre- and post-intervention effects, which will be reported elsewhere.

### Statistical analysis

Data analysis was performed using R (Version 4.4.0) and the package lavaan^86^. Group differences were tested using analysis of covariance^87^ within a regression analytic framework. Predictor variables included the dummy-coded treatment group (0 = WL, 1 = CBTd-E), the baseline measure of the outcome variable (T1), and dummy-coded center variables (with either one or two dummy-coded variables depending on whether the outcome data were available from two or three participating centers, respectively). Group differences were indicated by the significance of the regression coefficient associated with the treatment group predictor, thus reflecting baseline- and center-adjusted differences in units of the outcome measure. Cohen’s *d* was computed as an effect size measure, estimated from the treatment group regression coefficient and indicating the model-implied group differences. The confidence interval for Cohen’s *d* was derived from the confidence interval of the regression coefficient^88,89^. The standard deviation was based on all available data from the total group at baseline. For the outcome variables assessed at the 3-month post-assessment (T2), separate regression models were computed.

At baseline, missing data in outcome variables ranged from 2.13% to 31.91% in the CBTd-E group and from 0% to 40.43% in the WL group. At three months post-assessment (T2), missing data ranged from 21.28% to 46.8% in the CBTd-E group and from 10.64% to 42.55% in the WL group. At six months post-assessment (T3), missing data ranged from 31.91% to 53.19% in the CBTd-E group and 12.76% to 46.8% in the WL group. Missing data were caused by participant drop-out, organizational or technical issues with video recording for blinded assessment, patients refusing to participate in the video recording, and the fact that the EMA assessments were only conducted at two study centers (Hamburg and Marburg; see Fig. 1 for more information).

Missing data were handled using full information maximum likelihood estimation (FIML)^56,57^. This approach retained cases with and without missing values at 6-months post-assessment in the analysis, provided that the outcome was available at baseline. To test the stability of the findings across different approaches for handling missing data, two sensitivity analyses were conducted: (a) a single imputation using missForest^90^ and (b) a complete case analysis.

## Supplementary information

Supplement Efficacy of an emotion-oriented Cognitive Behavior Therapy for delusions (CBTd-E) compared to waitlist in a single-blinded randomized controlled trial

## Data Availability

Data from the study and all study materials will be available from the corresponding author after a reasonable request.

## Code availability

Code for the data analysis of the study will be available from the corresponding author after a reasonable request.
