# Supplementary material for: Efficacy of an emotion-oriented cognitive behavior therapy for delusions (CBTd-E) compared to waitlist in a single-blinded randomized-controlled trial
Source: Schizophrenia (Heidelb). 2026 Feb 17;12(1):29. doi: 10.1038/s41537-026-00737-y (PMC12992698; doi:10.1038/s41537-026-00737-y)
Supplement: Supplementary file 1 — Supplement Efficacy of an emotion-oriented Cognitive Behavior Therapy for delusions (CBTd-E) compared to waitlist in a single-blinded randomized controlled trial [file 41537_2026_737_MOESM1_ESM.pdf]

Supplement

Efficacy of an emotion-oriented Cognitive Behavior Therapy for delusions (CBTd-E) compared to waitlist in a single-blinded randomized-controlled trial

This supplement contains supporting information for the manuscript mentioned above. The results of complementary analyses are reported, and the main document discusses the findings.

**Table S1:**	Additional information, reliability, and validity of the measures	**3**
**Table S2:**	Sociodemographic and clinical characteristics of patients with psychotic disorders in the CBTd-E group who stayed in the trial (Per-Protocol Group) and patients who dropped out (Drop-Out-Group) a nd group comparison at baseline assessment.	**4**
**Table S3:**	Primary and secondary outcome variables and target mechanisms of patients with psychotic disorders in the CBTd-E group who stayed in the trial (Per-Protocol Group) and patients who dropped out (Drop-Out-Group) and group comparisons at baseline assessment.	**5**
**Table S4:**	Results of the linear regression analysis using single imputation as missing data strategy on effects of primary and secondary outcome variables and target mechanisms at 3 months post-assessment (missing values are imputed using single imputation missing forest)	**6**
**Table S5:**	Results of the linear regression analysis with listwise deletion on the primary and secondary outcome variables and target mechanisms at 3-months post-assessment	**7**
**Table S6**:	Results of the linear regression analysis using single imputation as missing data strategy on effects of primary and secondary outcome variables and target mechanisms at 6 months post-assessment (missing values are imputed using single imputation missing forest)	**8**
**Table S7**:	Results of the linear regression analysis with listwise deletion on the primary and secondary outcome variables and target mechanisms at 6-months post-assessment	**10**
**Table S8**:	Mean and standard deviations of the CBTd-E group and the Wait-list group at baseline, 3 months post-assessment, and 6 months post-assessment in the PSYRATS subscales	**12**
**Table S9**:	Results of the regression-based ANCOVA with Full Information Maximum Likelihood (FIML) for missing data handling at 6-months post-assessment (T3) on the primary and secondary outcome variables and target mechanisms in the intent-to-treat sample (ITT: *n* = 94), including additional subscales of the PSYRATS delusions scale (blind assessment)	**13**
**Table S10**:	Results of the linear regression analysis using single imputation as missing data strategy on effects of primary and secondary outcome variables and target mechanisms at 6-months post-assessment (missing values are imputed using single imputation missing forest), including additional subscales of the PSYRATS delusions scale (blind assessment)	**15**
**Table S11**:	Results of the linear regression analysis with listwise deletion on the Primary and secondary outcome variables and target mechanisms at 6-months post-assessment, including additional subscales of the PSYRATS delusions scale (blind assessment)	**18**

**Table S12**:	Moderator analysis (hierarchical linear regression analysis): does the Ability to regulate negative emotions moderate the association between the intervention group (CBTd-E vs. Wait list) and delusions at 6-months post-assessment, controlling for the baseline scores of delusions and study site and using FIML as missing value strategy	**19**
**Table S13**:	Moderator analysis (hierarchical linear regression analysis): does the Ability to regulate negative emotions moderate the association between intervention group (CBTd-E vs. Wait list) and delusions at 6-months post-assessment, controlling for the baseline scores of delusions and study site and using single imputation as missing value strategy	**20**
**Table S14:**	Moderator analysis (hierarchical linear regression analysis): does the Ability to regulate negative emotions moderate the association between intervention group (CBTd-E vs. Wait list) and delusions at 6-months post-assessment, controlling for the baseline scores of delusions and study site and using listwise deletion	**21**
**Table S15**:	Moderator analysis (hierarchical linear regression analysis): do negative self-schemata moderate the association between intervention group (CBTd-E vs. Wait list) and delusions at 6-months post-assessment, controlling for the baseline scores of delusions and study site and using FIML as missing value strategy	**22**
**Table S16**:	Moderator analysis (hierarchical linear regression analysis): do negative self-schemata moderate the association between intervention group (CBTd-E vs. Wait list) and delusions at 6-months post-assessment, controlling for the baseline scores of delusions and study site and using single imputation as missing value strategy	**23**
**Table S17:**	Moderator analysis (hierarchical linear regression analysis):do negative self-schemata moderate the association between intervention group (CBTd-E vs. Wait list) and delusions at 6-months post-assessment, controlling for the baseline scores of delusions and study site and using listwise deletion	**24**
**Table S18:**	Therapist competency rated with the CTS-RP	**25**
**References**		**26**

Table S1: Additional information on the items of the Ecological Momentary Assessment of Paranoid Delusions (EMA)

Ecological momentary assessment (EMA) was used to assess patients' state paranoia. Participants were asked to use an EMA device (Android smartphone provided by the study site) programmed with the movisens XS EMA application. At 10 random times a day over six consecutive days, patients were asked six questions regarding their state paranoia: three questions originated from the change-sensitive short-version of the Paranoia Checklist (Schlier et al., 2016) ("I need to be on my guard against others"; "Strangers and friends look at me critically"; "People try to upset me") and three questions were taken from previous EMA studies (Nittel et al., 2018) ("My thoughts are influenced by someone else"; "I feel others intend to harm me"; "I feel suspicious"). All items were answered on 7-point Likert scales, of which *the EMA mean score* was used (range: 1 - 7).

Table S2: Sociodemographic and clinical characteristics of patients with psychotic disorders in the CBTd-E group who stayed in the trial (Per-Protocol Group) and patients who dropped out (Drop-Out Group) and group comparison at baseline assessment (T1) .

Variable	Per-Protocol-Group (n = 34) *M (SD)/ n (%)*	Drop-Out group (n = 13) *M (SD)/ n (%)*	Test statistics	p
Sociodemographic variables				
Age (years)	38.68 (10.81)	35.69 (11.93)	t (45) = .82	.208
Gender				
Female	14 (41.18 %)	2 (15.38 %)	Chi^2 (1) = 2.786	.168
Male	20 (58.82 %)	11 (84.62 %)		
Education grade[1]				
9 school years [1]:	7 (20.59 %)	2 (15.38 %)	Chi^2 (2) = 0.172	.918
10 school years [2]:	7 (20.59 %)	3 (23.08 %)		
High school equivalent:	20 (58.82 %)	8 (61.54 %)		
Estimated verbal IQ	103.93 (13.43)[1]	107.86 (13.23)[2]	t (35) = .69	.490
Family status:				
single	27 (79.42 %)	10 (76.92 %)	Chi^2 (3) = 4.533	.209
in relationship	4 (11.76 %)	0 (0 %)		
married	2 (5.88 %)	3 (23.08 %)		
divorced	1 (2.94%)	0 (0 %)		
Living situation:				
Living alone:	15 (44.12 %)	7 (53.85 %)	Chi^2 (4) = 1.371	.849
Living with partner:	6 (17.65 %)	2 (15.38 %)		
Living with parents:	4 (11.76 %)	1 (7.69 %		
Shared flat:	3 (8.82 %)	2 (15.38 %)		
Assisted living:	6 (17.65 %)	1 (7.69 %)		
Clinical variables				
Main diagnosis:				
Schizophrenia	25 (73.53 %)	11 (84.62%)	Chi^2 (4) = 4.539	.338
Schizoaffective disorder	6 (17.65 %)	1 (7.69 %)		
Delusional disorder	0 (0 %)	1 (7.69 %)		
Schizotypical personality disorder	2 (5.88 %)	0 (0%)		
Brief psychotic disorder	1 (2.94%)	0 (0%)		
Medication at baseline	32 (94.12 %)	11 (84.62%)	Chi^2 (1) = 1.09	.296
Antipsychotic medication	29 (85.29 %)	11 (84.62 %)	Chi^2 (1) = .003	.953
Antidepressive medication	4 (11.76%)	2 (15.38 %)	Chi^2 (1) = .11	.739
Duration of psychotic disorder	15.38 (10.90)	12.77 (13.14)	t (45) = -.69	.245

Notes: M = Mean; SD = Standard deviation; [1]German Hauptschulabschluss (9 school years), [2] = German Realschulabschluss (10 school years), [1] = n = 30, [2] n = 7,

Table S3. Primary and secondary outcome variables and target mechanisms of patients with psychotic disorders in the CBTd-E group who stayed in the trial (Per-Protocol Group) and patients who dropped out (Drop-Out-Group) and group comparisons at baseline assessment

Variable	Per-Protocol-Group (*n* = 34) *M (SD)/ n (%)*		Drop-Out group (*n* = 13) *M (SD)/ n (%)*		Test statistics	*p*
Primary outcome variables						
PSYRATS delusions scale (Blind)	13.35 (5.37)	26	9.71 (4.89)	7	*t*(31) = -1.61	.116
PSYRATS delusions scale (Unblinded)	14.85 (3.53)	34	15.17 (3.79)	12	*t*(44)= .260	.798
Secondary outcome variables						
PDI-21 grand total score (SR)	65.23 (55.09)	32	77.25 (60.48)	12	*t*(42) = 0.63	.533
…EMA persecutory delusions (SR)	2.69 (1.35)	27	1.62 (0.97)	5	*t*(30) = -1.68	.104
PANSS POS (Blind)	16.16 (3.60)	25	15.14 (5.15)	7	*t*(30) = -0.58	.567
PANSS POS (Unblinded)	17.06 (3.30)	34	16.25 (3.52)	12	*t*(44) = -0.72	.116
PANSS NEG (Blind)	16.64 (3.60)	25	15.86 (4.41)	7	*t*(30) = -0.58	.632
PANSS NEG (Unblinded)	16.12 (5.17)	34	15.00 (3.36)	12	*t*(44) = -0.70	.490
PANSS GEN (Blind)	36.60 (6.44)	25	15.86 (4.14)	7	*t*(30) = -0.77	.450
PANSS GEN (Unblinded)	33.56 (6.74)	34	33.33 (7.14)	12	*t*(44) =-0.10	.922
CDSS total score (Blind)	6.18 (3.92)	25	7.66 (3.85)	7	*t*(30) = 0.89	.382
CDSS total score (Unblinded)	6.50 (4.50)	34	8.33 (4.83)	12	*t(*44) = 1.19	.240
RFS general functioning (Blind)	6.80 (2.24)	25	7.71 (1.15)	7	*t*(30) = 1.04	.309
RFS general functioning (Unblinded)	6.44 (2.38)	34	6.58 (2.32)	12	*t(*44) = 0.18	.859
RFS social functioning (Blind)	7.54 (2.08)	25	7.14 (2.85)	7	*t(*30) = -0.41	.683
RFS social functioning (Unblinded)	6.79 (2.13)	34	6.67 (2.84)	12	*t(*44) =-0.16	.871
CPZ	722.84 (473.15)	33	506.63 (462.71)	13	*t*(44) = -0.14	.167
PSYRATS voices scale (Blind)	11.19 (14.02)	26	0.57 (1.51)	7	*t*(31) = -1.98	.057
PSYRATS voices scale (Unblinded)	11.32 (14.02)	34	10.58 (15.69)	11	*t*(44) = -0.16	.880
Target mechanisms						
ERQ expressive suppression (SR)	13.84 (5.55)	31	14.08 (3.45)	12	*t*(41) = 0.14	.888
ERQ reappraisal (SR)	15.48 (6.64)	31	17.33 (6.69)	12	*t*(41) = 0.82	.418
ERSQ total score (SR)	54.90 (18.28)	31	47.58 (20.65)	12	*t*(41) = 0.73	.469
ERI positive emotions (SR)	23.59 (10.96)	32	18.14 (7.73)	12	*t*(42) = -1.58	.123
ERI negative emotions (SR)	39.38 (12.45)	32	41.50 (10.61)	12	*t*(42) = 0.52	.605
PSWQ sum score (SR)	54.10 (14.36)	32	50.42 (9.43)	12	*t*(42) = -0.82	.416
ISI insomnia sum score (SR)	11.55 (6.48)	31	13.08 (5.26)	12	*t*(41) = 0.731	.469
RSES sum score (SR)	16.19 (7.05)	32	14.83 (7.30)	12	*t(*42) = -0.57	.575
SCS total score (SR)	48.27 (14.17)	32	44.67 (14.91)	12	*t*(42) = -0.74	.463
BCSS negative-self (SR)	9.35 (5.71)	31	8.58 (6.23)	12	*t*(41) = -0.39	.703
BCSS positive-self (SR)	12.32 (4.71)	31	12.00 (6.03)	12	*t*(41) = -0.19	.853
BCSSS negative-others (SR)	10.08 (5.00)	31	7.33 (7.00)	12	*t*(41) = -1.44	.157
BCSS positive-others (SR)	12.34 (4.26)	31	13.42 (5.09)	12	*t*(41) = 0.70	.486

Notes: * p ≤ .05; ** p ≤ .01; SR = Self-rating; PSYRATS = Psychotic Rating Symptom Scale; PDI = Peters et al. Delusions Inventory; EMA = ecological momentary assessment of persecutory delusions over 6 consecutive days and 10 time points: mean score; PANSS = Positive and Negative Syndrome Scale; PANSS POS = PANSS positive scale; PANSS NEG = PANSS negative scale; PANSS GEN = PANSS general psychopathology scale; CDSS = Calgary Depression Rating Scale; RFS = Role functioning scale: CPZ = Chlorpromazine equivalent of antipsychotic medication, ERQ = Emotion Regulation Questionnaire; ERSQ = Emotion Regulation Style Questionnaire; ERI = Emotion Regulation Inventory; PSWQ = Penn State Worry Questionnaire; ISI = Insomnia Severity Index; RSE = Rosenberg Self-Esteem Scale; SCS = Self-Compassion Scale; BCSS = Brief Core Schema Scale.

Table S4: Results of the linear regression analysis using single imputation as missing data strategy on effects of primary and secondary outcome variables and target mechanisms at 3-months post-assessment (missing values are imputed using single imputation missing forest)

		Group differences			Effect size	
Measure	*n (analysis)*	*beta* $_{unst.}$[1.]	*SE*	*p*	*d*	*95% CI*
Primary outcome variables						
PSYRATS delusions scale (Blind)	94	-0.421	0.886	.636	-0.103	[-0.533; 0.327]
PSYRATS delusions scale (Unblinded)	94	-1.604	1.234	.197	-0.458	[-1.158; 0.242]
Secondary outcome variables						
PDI-21 grand total score (SR)	94	-8.942	7.897	.261	-0.162	[-0.446; 0.122]
…EMA persecutory delusions (SR)	94	0.092	0.167	.581	0.074	[-0.191; 0.339]
CPZ	94	14.661	48.115	.761	0.029	[-0.162; 0.221]
PSYRATS voices scale (Blind)	94	1.691	1.046	.110	0.154	[-0.035; 0.344]
PSYRATS voices scale (Unblinded)	94	0.555	1.550	.721	0.042	[-0.190; 0.273]
Target mechanisms						
ERQ expressive suppression (SR)	94	2.282	0.900	.013 *	0.428	[0.093; 0.763]
ERQ reappraisal (SR)	94	3.351	1.322	.013 *	0.467	[0.101; 0.833]
ERSQ total score (SR)	94	-2.358	2.610	.369	-0.136	[-0.436; 0.164]
ERI positive emotions (SR)	94	-2.076	1.429	.150	-0.211	[-0.500; 0.078]
ERI negative emotions (SR)	94	0.140	1.844	.940	0.013	[-0.331; 0.357]
PSWQ sum score (SR)	94	-7.707	2.413	.002 **	-0.628	[-1.019; -0.238]
ISI insomnia sum score (SR)	94	-2.587	0.877	.004 **	-0.430	[-0.720; -0.140]
RSES sum score (SR)	94	1.238	0.804	.118	0.187	[-0.049; 0.423]
SCS total score (SR)	94	3.251	2.147	.134	0.211	[-0.066; 0.489]
BCSS negative-self (SR)	94	0.291	0.706	.681	0.052	[-0.200; 0.305]
BCSS positive-self (SR)	94	-0.780	0.786	.324	-0.149	[-0.446; 0.149]
BCSSS negative-others (SR)	94	-1.088	0.891	.225	-0.195	[-0.512; 0.122]
BCSS positive-others (SR)	94	0.511	0.798	.523	0.122	[-0.255; 0.499]

Notes: Regression analysis of the post-scores (dependent variable) on group (independent variable: 0 = WL, 1 = CBTd-E) while controlling for study site; * $p \leq .05$; ** $p \leq .01$; [1] = unstandardized beta; SE = Standard error; SR = Self-rating; PSYRATS = Psychotic Rating Symptom Scales; PDI = Peters et al. Delusions Inventory; EMA = ecological momentary assessment of persecutory delusions over 6 consecutive days and 10 time points: mean score; CPZ = chlorpromazine equivalent of antipsychotic medication; ERQ = Emotion Regulation Questionnaire; ERSQ = Emotion Regulation Style Questionnaire; ERI = Emotion Regulation Inventory; PSWQ = Penn State Worry Questionnaire; ISI = Insomnia Severity Index; RSE = Rosenberg Self-Esteem Scale; SCS = Self-Compassion Scale; BCSS = Brief Core Schema Scale.

Table S5: Results of the linear regression analysis with listwise deletion on the primary and secondary outcome variables and target mechanisms at 3-months post-assessment

Measure	*n (analysis)*	Group differences *beta* $_{unst.}$[1.]	*SE*	*p*	Effect size *d*	*95% CI*
Primary outcome variables						
PSYRATS delusions scale (Blind)	48	-1.423	1.429	.325	-0.300	[-0.906; 0.307]
PSYRATS delusions scale (Unblinded)	79	-1.665	1.434	.249	-0.473	[-1.285; 0.339]
Secondary outcome variables						
PDI-21 grand total score (SR)	66	-6.106	9.909	.540	-0.107	[-0.456; 0.241]
…EMA persecutory delusions (SR)	51	0.295	0.276	.290	0.212	[-0.186; 0.609]
CPZ	79	6.860	57.001	.905	0.014	[-0.212; 0.240]
PSYRATS voices scale (Blind)	48	2.240	1.429	.124	0.188	[-0.054; 0.431]
PSYRATS voices scale (Unblinded)	79	1.052	1.744	.548	0.079	[-0.182; 0.340]
Target mechanisms						
ERQ expressive suppression (SR)	66	2.739	1.262	.034 *	0.501	[0.040; 0.962]
ERQ reappraisal (SR)	65	5.379	1.891	.006 **	0.723	[0.215; 1.232]
ERSQ total score (SR)	67	-4.378	3.541	.221	-0.248	[-0.649; 0.153]
ERI positive emotions (SR)	67	-3.019	1.858	.109	-0.299	[-0.667; 0.069]
ERI negative emotions (SR)	67	1.260	2.522	.619	0.115	[-0.346; 0.576
PSWQ sum score (SR)	66	-10.942	3.257	.001 **	-0.869	[-1.386; -0.352]
ISI insomnia sum score (SR)	65	-3.156	1.106	.006 **	-0.506	[-0.860; -0.151]
RSES sum score (SR)	67	1.533	1.091	.165	0.222	[-0.094; 0.537]
SCS total score (SR)	65	4.415	2.911	.135	0.279	[-0.089; 0.647]
BCSS negative-self (SR)	65	0.473	0.926	.612	0.083	[-0.244; 0.410]
BCSS positive-self (SR)	65	-0.836	1.076	.440	-0.156	[-0.556; 0.245]
BCSSS negative-others (SR)	64	-1.034	1.162	.377	-0.179	[-0.581; 0.223]
BCSS positive-others (SR)	65	0.522	1.130	.646	0.120	[-0.400; 0.641]

Notes: Regression analysis of the post-scores (dependent variable) on group (independent variable: 0 = WL, 1 = CBTd-E) while controlling for study site; * $p \leq .05$; ** $p \leq .01$; [1] = unstandardized beta; SE = Standard error; SR = Self-rating; PSYRATS = Psychotic Rating Symptom Scales; PDI = Peters et al. Delusions Inventory; EMA = ecological momentary assessment of persecutory delusions over 6 consecutive days and 10 time points: mean score; CPZ = chlorpromazine equivalent of antipsychotic medication; ERQ = Emotion Regulation Questionnaire; ERSQ = Emotion Regulation Style Questionnaire; ERI = Emotion Regulation Inventory; PSWQ = Penn State Worry Questionnaire; ISI = Insomnia Severity Index; RSE = Rosenberg Self-Esteem Scale; SCS = Self-Compassion Scale; BCSS = Brief Core Schema Scale.

Table S6: Results of the linear regression analysis using single imputation as missing data strategy on effects of primary and secondary outcome variables and target mechanisms at 6-months post-assessment (missing values are imputed using single imputation missing forest)

		Group differences			Effect size	
Measure	*n* (analysis)	*beta* $_{unst.}$ [1.]	*SE*	*p*	*d*	*95% CI*
Primary outcome variables						
PSYRATS delusions scale (Blind)	94	-1.354	1.016	.186	-0.331	[-0.824; 0.162]
PSYRATS delusions scale (Unblinded)	94	-1.681	1.148	.146	-0.480	[-1.132; 0.171]
Secondary outcome variables						
PDI-21 grand total score (SR)	94	-8.710	6.771	.231	-0.148	[-0.392; 0.096]
…EMA persecutory delusions (SR)	94	0.027	0.146	.856	0.021	[-0.211; 0.254]
PANSS POS (Blind)	94	-0.656	0.600	.278	-0.171	[-0.481; 0.140]
PANSS POS (Unblinded)	94	-1.415	0.679	.040 *	-0.380	[-0.743; -0.018]
PANSS NEG (Blind)	94	-0.116	0.685	.866	-0.030	[-0.386; 0.325]
PANSS NEG (Unblinded)	94	-0.325	0.766	.672	-0.069	[-0.389; 0.252]
PANSS GEN (Blind)	94	-2.172	1.024	.037 *	-0.372	[-0.720; -0.024]
PANSS GEN (Unblinded)	94	-2.624	1.207	.032 *	-0.409	[-0.783; -0.035]
CDSS total score (Blind)	94	-0.874	0.599	.148	-0.277	[-0.653; 0.100]
CDSS total score (Unblinded)	94	-1.768	0.571	.003**	-0.416	[-0.683; -0.149]
RFS general functioning (Blind)	94	0.099	0.305	.745	0.052	[-0.267; 0.372]
RFS general functioning (Unblinded)	94	0.704	0.395	.078	0.278	[-0.032; 0.587]
RFS social functioning (Blind)	94	0.446	0.344	.197	0.238	[-0.126; 0.602]
RFS social functioning (Unblinded)	94	0.785	0.411	.059	0.338	[-0.014; 0.690]
CPZ	94	-83.343	122.143	.497	-0.167	[-0.652; 0.319]
PSYRATS voices scale (Blind)	94	-1.512	1.521	.323	-0.138	[-0.414; 0.138]
PSYRATS voices scale (Unblinded)	94	-2.465	1.705	.152	-0.185	[-0.440; 0.069]
Target mechanisms						
ERQ expressive suppression (SR)	94	0.951	0.722	.191	0.178	[-0.091; 0.447]
ERQ reappraisal (SR)	94	2.549	0.980	.011 *	0.355	[0.084; 0.627]
ERSQ total score (SR)	94	-0.401	2.727	.883	-0.023	[-0.337; 0.290]
ERI positive emotions (SR)	94	-2.896	1.396	.041 *	-0.294	[-0.577; -0.012]
ERI negative emotions (SR)	94	-2.423	1.925	.211	-0.227	[-0.586; 0.132]
PSWQ sum score (SR)	94	-4.527	2.360	.058	-0.369	[-0.751; 0.013]
ISI insomnia sum score (SR)	94	-2.673	0.938	.005 **	-0.444	[-0.754; -0.135]
RSES sum score (SR)	94	1.948	0.833	.022 *	0.288	[0.043; 0.533]
SCS total score (SR)	94	1.479	2.056	.474	0.096	[-0.169; 0.362]
BCSS negative-self (SR)	94	-0.348	0.736	.638	-0.063	[-0.326; 0.201]

BCSS positive-self (SR)	94	0.248	0.819	.763	0.047	[-0.263; 0.357]
BCSSS negative-others (SR)	94	-0.905	0.862	.297	-0.162	[-0.469; 0.145]
BCSS positive-others (SR)	94	0.222	0.810	.784	0.053	[-0.330; 0.435]

Notes: Regression analysis of the post-scores (dependent variable) on group (independent variable: 0 = WL, 1 = CBTd-E) while controlling for study site; * $p \leq .05$; ** $p \leq .01$; [1] = unstandardized beta; *SE* = Standard error; SR = Self-rating; PSYRATS = Psychotic Rating Symptom Scales; PDI = Peters et al. Delusions Inventory; EMA = ecological momentary assessment of persecutory delusions over 6 consecutive days and 10 time points: mean score; PANSS = Positive and Negative Syndrome Scale; PANSS POS = PANSS positive scale; PANSS NEG = PANSS negative scale; PANSS GEN = PANSS general psychopathology scale; CDSS = Calgary Depression Rating Scale; RFS = Role Functioning Scale; CPZ = chlorpromazine equivalent of antipsychotic medication; ERQ = Emotion Regulation Questionnaire; ERSQ = Emotion Regulation Style Questionnaire; ERI = Emotion Regulation Inventory; PSWQ = Penn State Worry Questionnaire; ISI = Insomnia Severity Index; RSE = Rosenberg Self-Esteem Scale; SCS = Self-Compassion Scale; BCSS = Brief Core Schema Scale.

Table S7: Results of the linear regression analysis with listwise deletion on the primary and secondary outcome variables and target mechanisms at 6-months post-assessment

Measure	*n* (analysis)	Group differences *beta* unst.[1.]	*SE*	*p*	Effect size *d*	*95% CI*
Primary outcome variables						
PSYRATS delusions scale (Blind)	42	-2.145	2.056	.303	-0.451	[-1.327; 0.424]
PSYRATS delusions scale (Unblinded)	72	-2.236	1.488	.138	-0.635	[-1.479; 0.208]
Secondary outcome variables						
PDI-21 grand total score (SR)	62	-8.548	9.515	.373	-0.150	[-0.485; 0.185]
…EMA persecutory delusions (SR)	48	-0.154	0.249	.539	-0.110	[-0.470; 0.249]
PANSS POS (Blind)	47	-1.527	1.049	.153	-0.341	[-0.812; 0.131]
PANSS POS (Unblinded)	70	-1.760	0.872	.048 *	-0.470	[-0.936; -0.005]
PANSS NEG (Blind)	47	0.034	1.331	.980	0.007	[-0.582; 0.597]
PANSS NEG (Unblinded)	70	-0.536	1.014	.599	-0.112	[-0.537; 0.312]
PANSS GEN (Blind)	47	-3.755	1.888	.053	-0.558	[-1.124; 0.008]
PANSS GEN (Unblinded)	70	-3.018	1.593	.063	-0.468	[-0.962; 0.025]
CDSS total score (Blind)	47	-1.074	1.126	.345	-0.291	[-0.907; 0.325]
CDSS total score (Unblinded)	69	-2.301	0.729	.002 **	-0.540	[-0.881; -0.198]
RFS general functioning (Blind)	46	0.321	0.616	.605	0.147	[-0.422; 0.716]
RFS general functioning (Unblinded)	68	1.048	0.526	.051	0.411	[-0.001; 0.824]
RFS social functioning (Blind)	46	0.855	0.617	.173	0.391	[-0,178; 0.961]
RFS social functioning (Unblinded)	68	0.868	0.557	.124	0.372	[-0.105; 0.848]
CPZ	68	-123.283	171.588	.475	-0.245	[-0.928; 0.437]
PSYRATS voices scale (Blind)	42	-2.895	3.086	.354	-0.244	[-0.769; 0.282]
PSYRATS voices scale (Unblind)	72	-1.822	2.112	.391	-0.137	[-0.453; 0.180]
Target mechanisms						
ERQ expressive suppression (SR)	62	1.198	1.105	.283	0.219	[-0.186; 0.624]
ERQ reappraisal (SR)	61	4.371	1.452	.004 **	0.588	[0.197; 0.978]]
ERSQ total score (SR)	63	0.282	3.916	.943	0.016	[-0.428; 0.460]
ERI positive emotions (SR)	63	-3.557	1.977	.077	-0.352	[-0.744; 0.040]
ERI negative emotions (SR)	63	-2.541	2.893	.384	-0.232	[-0.762; 0.297]
PSWQ sum score (SR)	63	-6.443	3.450	.067	-0.512	[-1.059; 0.036]
ISI insomnia sum score (SR)	62	-3.074	1.304	.022 *	-0.493	[-0.911; -0.074]
RSES sum score (SR)	63	2.517	1.212	.042 *	0.364	[0.013; 0.715]
SCS total score (SR)	62	1.162	3.019	.702	0.073	[-0.308; 0.455]

BCSS negative-self (SR)	62	-0.073	1.069	.946	-0.013	[-0.391; 0.365]
BCSS positive-self (SR)	62	0.054	1.215	.964	0.010	[-0.443; 0.463]
BCSSS negative-others (SR)	61	-1.297	1.161	.269	-0.224	[-0.626; 0.178]
BCSS positive-others (SR)	62	-0.049	1.208	.968	-0.011	[-0.568; 0.546]

Notes: Regression analysis of the post-scores (dependent variable) on group (independent variable: 0 = WL, 1 = CBTd-E) while controlling for study site; * $p \leq .05$; ** $p \leq .01$; [1] = unstandardized beta; SE = Standard error; SR = Self-rating; PSYRATS = Psychotic Rating Symptom Scales; PDI = Peters et al. Delusions Inventory; EMA = ecological momentary assessment of persecutory delusions over 6 consecutive days and 10 time points: mean score; PANSS = Positive and Negative Syndrome Scale; PANSS POS = PANSS positive scale; PANSS NEG = PANSS negative scale; PANSS GEN = PANSS general psychopathology scale; CDSS = Calgary Depression Rating Scale; RFS = Role Functioning Scale; CPZ = chlorpromazine equivalent score of antipsychotic medication; ERQ = Emotion Regulation Questionnaire; ERSQ = Emotion Regulation Style Questionnaire; ERI = Emotion Regulation Inventory; PSWQ = Penn State Worry Questionnaire; ISI = Insomnia Severity Index; RSE = Rosenberg Self-Esteem Scale; SCS = Self-Compassion Scale; BCSS = Brief Core Schema Scale.

Table S8: Mean and standard deviations of the CBTd-E group and the Wait-list group at baseline, 3 months post-assessment, and 6 months post-assessment in the PSYRATS subscales

Measure	CBTd-E group Baseline (T1) Mean (SD)	*n*	WL group Baseline (T1) Mean (SD)	n	CBTd-E group 3-months post-assessment (T2) Mean (SD)	n	WL group 3-months post-assessment (T2) Mean (SD)	*n*	CBTd-E group 6-months post-assessment (T3) Mean (SD)	*n*	WL group 6 months-post-assessment (T3) Mean (SD)	*n*
PSYRATS delusions scale: Subscales:												
Amount of preoccupation (Blind)	1.64 (1.14)	33	2.00 (0.94)	28	1.23 (0.88)	31	1.38 (0.82)	29	1.23 (1.11)	22	1.37 (1.08)	27
Duration of preoccupation (Blind)	2.09 (1.10)	33	2.54 (0.96)	28	2.16 (1.19)	31	1.97 (1.12)	29	1.41 (1.14)	22	2.19 (1.18)	27
Conviction (Blind)	2.27 (1.38)	33	2.68 (0.86)	28	2.06 (1.46)	31	2.48 (1.27)	29	2.00 (1.48)	22	2.26 (1.58)	27
Amount of distress (Blind)	2.73 (1.48)	33	3.21 (0.96)	28	2.29 (1.40)	31	2.69 (1.51)	29	1.86 (1.73)	22	2.41 (1.57)	27
Intensity of distress (Blind)	2.64 (1.41)	33	2.75 (0.89)	28	1.90 (1.47)	31	2.31 (1.37)	29	1.41 (1.37)	22	2.00 (1.18)	27
Disruption of daily life (Blind)	1.21 (0.86)	33	1.61 (0.79)	28	0.81 (0.95)	31	1.38 (0.82)	29	0.82 (1.01)	22	1.19 (0.96)	27
PSYRATS delusions scale: Subscales:												
Amount of preoccupation (Unblinded)	2.17 (1.10)	46	2.21 (0.93)	47	1.19 (1.08)	37	1.50 (1.21)	42	1.31 (1.15)	32	1.54 (1.12)	41
Duration of preoccupation (Unblinded)	2.54 (0.98)	46	2.57 (0.99)	47	1.78 (1.34)	37	1.76 (1.32)	42	1.56 (1.13)	32	2.02 (1.31)	41
Conviction (Unblinded)	2.72 (1.07)	46	2.83 (1.03)	47	1.84 (1.54)	37	2.21 (1.47)	42	2.03 (1.36)	32	2.05 (1.52)	41
Amount of distress (Unblinded)	3.13 (0.93)	46	3.15 (1.04)	47	1.89 (1.58)	37	2.24 (1.72)	42	2.00 (1.57)	32	2.37 (1.58)	41
Intensity of distress (Unblinded)	2.61 (0.95)	46	2.66 (0.89)	47	1.59 (1.36)	37	1.93 (1.50)	42	1.63 (1.31)	32	2.17 (1.36)	41
Disruption of daily life (Unblinded)	1.76 (0.85)	46	1.91 (0.86)	47	1.03 (1.07)	37	1.36 (1.12)	42	0.94 (0.98)	32	1.51 (1.08)	41

Notes: WL = Wait-list; SD = Standard deviation; Blind = Blind assessment; Unblinded = Unblinded assessment; PSYRATS = Psychotic Rating Symptom Scale.

Table S9: Results of the regression-based ANCOVA with Full Information Maximum Likelihood (FIML) for missing data handling at 6-months post-assessment (T3) on the primary and secondary outcome variables and target mechanisms in the intent-to-treat sample (ITT: *n* = 94), including additional subscales of the PSYRATS delusions scale (blind assessment)

		Group differences			Effect size	
Measure	*n (analysis)*	*beta* unst.[1]	*SE*	*p*	*d*	*95% CI*
Primary outcome variables						
PSYRATS delusions scale (Blind)	61	-2.145	1.955	.273	-0.451	[-1.258; 0.355]
PSYRATS delusions: amount of preoccupation	61	-0.044	0.314	.889	-0.041	[-0.621; 0.538]
PSYRATS delusions: duration of preoccupation	61	-0.871	0.365	.017*	-0.826	[-1.504; -0.147]
PSYRATS delusions: conviction	61	-0.163	0.474	.731	-0.138	[-0.928; 0.651]
PSYRATS delusions: amount of distress	61	-0.246	0.509	.629	-0.192	[-0.968; 0.585]
PSYRATS delusions: intensity of distress	61	-0.352	0.393	.372	-0.295	[-0.943; 0.352]
PSYRATS delusions: disruption of daily life	61	-0.343	0.285	.229	-0.407	[-1.070; 0.256]
PSYRATS delusions scale (Unblinded)	93	-2.236	1.435	.119	-0.635	[-1.435; 0.164]
Secondary outcome variables						
PDI-21 grand total score (SR)	88	-8.548	9.123	.349	-0.150	[-0.464; 0.164]
…EMA persecutory delusions (SR)	72	-0.154	0.239	.518	-0.110	[-0.446; 0.225]
PANSS POS (Blind)	62	-1.527	1.004	.128	-0.341	[-0.779; 0.098]
PANSS POS (Unblinded)	93	-1.760	0.840	.036 *	-0.470	[-0.910; -0.030]
PANSS NEG (Blind)	62	0.034	1.273	.979	0.007	[-0.541; 0.556]
PANSS NEG (Unblinded)	93	-0.536	0.977	.583	-0.112	[-0.514; 0.289]
PANSS GEN (Blind)	62	-3.755	1.806	.038*	-0.558	[-1.084; -0.032]
PANSS GEN (Unblinded)	93	-3.018	1.535	.049*	-0.468	[-0.935; -0.002]
CDSS total score (Blind)	62	-1.074	1.077	.319	-0.291	[-0.864; 0.281]
CDSS total score (Unblinded)	93	-2.301	0.702	.001 **	-0.540	[-0.862; -0.217]
RFS general functioning (Blind)	61	0.321	0.589	.586	0.147	[-0.381; 0.675]
RFS general functioning (Unblinded)	93	1.048	0.506	.038*	0.411	[0.022; 0.801]
RFS social functioning (Blind)	61	0.855	0.590	.147	0.391	[-0.137; 0.920]
RFS social functioning (Unblinded)	93	0.868	0.536	.105	0.372	[-0.078; 0.822]
CPZ	93	-123.283	165.160	.455	-0.245	[-0.890; 0.399]
PSYRATS voices scale (Blind)	61	-2.895	2.935	.324	-0.244	[-0.727; 0.240]
PSYRATS voices scale (Unblinded)	93	-1.822	2.037	.371	-0.137	[-0.436; 0.163]
Target mechanisms						
ERQ expressive suppression (SR)	88	1.198	1.060	.258	0.219	[-0.161; 0.599]

ERQ reappraisal (SR)	87	4.371	1.391	.002 **	0.588	[0.221; 0.954]]
ERSQ total score (SR)	88	0.282	3.758	.940	0.016	[-0.401; 0.433]
ERI positive emotions (SR)	89	-3.557	1.897	.061	-0.352	[-0.720; 0.016]
ERI negative emotions (SR)	89	-2.541	2.776	.360	-0.232	[-0.730; 0.265]
PSWQ sum score (SR)	89	-6.556	3.347	.050 *	-0.520	[-1.041; 0.000]
ISI insomnia sum score (SR)	87	-3.074	1.250	.014 *	-0.493	[-0.885; -0.100]
RSES sum score (SR)	89	2.517	1.163	.030 *	0.364	[0.034; 0.693]
SCS total score (SR)	88	1.162	2.895	.688	0.073	[-0.285; 0.432]
BCSS negative-self (SR)	88	-0.073	1.025	.944	-0.013	[-0.367; 0.342]
BCSS positive-self (SR)	88	0.054	1.165	.963	0.010	[-0.415; 0.436]
BCSSS negative-others (SR)	87	-1.297	1.112	.243	-0.224	[-0.601; 0.153]
BCSS positive-others (SR)	88	-0.049	1.158	.966	-0.011	[-0.534; 0.511]

Notes: Regression analysis of the post-scores (dependent variable) on group (independent variable: 0 = WL, 1 = CBTd-E) while controlling for treatment center; PSYRATS delusions subscales printed in bold; * : * $p \leq .05$; ** $p \leq .01$; [1] = unstandardized beta; SE = Standard error; SR = Self-rating; PSYRATS = Psychotic Rating Symptom Scales; PDI = Peters et al. Delusions Inventory; EMA = ecological momentary assessment of persecutory delusions over 6 consecutive days and 10 time points: mean score; PANSS = Positive and Negative Syndrome Scale; PANSS POS = PANSS positive scale; PANSS NEG = PANSS negative scale; PANSS GEN = PANSS general psychopathology scale; CDSS = Calgary Depression Rating Scale; RFS = Role Functioning Scale; CPZ = chlorpromazine equivalent of antipsychotic medication; ERQ = Emotion Regulation Questionnaire; ERSQ = Emotion Regulation Style Questionnaire; ERI = Emotion Regulation Inventory; PSWQ = Penn State Worry Questionnaire; ISI = Insomnia Severity Index; RSE = Rosenberg Self-Esteem Scale; SCS = Self-Compassion Scale; BCSS = Brief Core Schema Scale.

Table S10: Results of the linear regression analysis using single imputation as missing data strategy on effects of primary and secondary outcome variables and target mechanisms at 6 months post-assessment (missing values are imputed using single imputation missing forest), including additional subscales of the PSYRATS delusions scale (blind assessment)

		Group differences			Effect size	
Measure	*n* (analysis)	*beta* $_{unst.}$ [1.]	*SE*	*p*	*d*	*95% CI*
Primary outcome variables						
PSYRATS delusions scale (Blind)	94	-1.282	0.999	.203	-0.319	[-0.811; 0.174]
PSYRATS delusions: amount of preoccupation	94	-0.073	0.165	.658	-0.083	[-0.456; 0.289]
PSYRATS delusions: duration of preoccupation	94	-0.413	0.188	.031*	-0.460	[-0.875; -0.044]
PSYRATS delusions: conviction	94	-0.126	0.239	.599	-0.130	[-0.620; 0.360]
PSYRATS delusions: amount of distress	94	-0.258	0.265	.333	-0.242	[-0.737; 0.253]
PSYRATS delusions: intensity of distress	94	-0.340	0.199	.092	-0.337	[-0.730; 0.056]
PSYRATS delusions: disruption of daily life	94	-0.178	0.154	.249	-0.254	[-0.689; 0.181]
PSYRATS delusions scale (Unblinded)	94	-1.651	1.144	.152	-0.471	[-1.120; 0.177]
Secondary outcome variables						
PDI-21 grand total score (SR)	94	-8.895	6.810	.195	-0.161	[-0.407; 0.084]
…EMA persecutory delusions (SR)	94	0.051	0.146	.729	0.040	[-0.191; 0.271]
PANSS POS (Blind)	94	-0.654	0.601	.280	-0.172	[-0.486; 0.142]
PANSS POS (Unblinded)	94	-1.395	0.684	.044 *	-0.375	[-0.740; -0.010]
PANSS NEG (Blind)	94	0.049	0.682	.942	0.013	[-0.343; 0.369]
PANSS NEG (Unblinded)	94	-0.305	0.763	.690	-0.064	[-0.384; 0.255]
PANSS GEN (Blind)	94	-2.146	1.024	.039 *	-0.370	[-0.721; -0.019]
PANSS GEN (Unblinded)	94	-2.558	1.216	.038 *	-0.399	[-0.775; -0.022]
CDSS total score (Blind)	94	-0.887	0.597	.141	-0.284	[-0.663; 0.096]
CDSS total score (Unblinded)	94	-1.803	0.577	.002**	-0.424	[-0.694; -0.155]
RFS general functioning (Blind)	94	0.098	0.302	.746	0.053	[-0.269; 0.375]
RFS general functioning (Unblinded)	94	0.687	0.396	.086	0.271	[-0.040; 0.582]
RFS social functioning (Blind)	94	0.495	0.349	.159	0.270	[-0.108; 0.648]
RFS social functioning (Unblinded)	94	0.825	0.414	.049 *	0.355	[0.001; 0.710]
CPZ	94	-63.547	122.451	.605	-0.127	[-0.614; 0.360]
PSYRATS voices scale (Blind)	94	-1.203	1.558	.442	-0.111	[-0.396; 0.175]
PSYRATS voices scale (Unblinded)	94	-2.512	1.734	.151	-0.189	[-0.448; 0.070]
Target mechanisms						
ERQ expressive suppression (SR)	94	0.866	0.725	.236	0.163	[-0.108; 0.434]
ERQ reappraisal (SR)	94	2.505	0.981	.012 *	0.349	[0.077; 0.621]
ERSQ total score (SR)	94	-0.386	2.762	.889	-0.022	[-0.342; 0.297]

ERI positive emotions (SR)	94	-2.512	1.410	.078	-0.255	[-0.540; -0.029]
ERI negative emotions (SR)	94	-2.575	1.938	.187	-0.242	[-0.603; 0.120]
PSWQ sum score (SR)	94	-4.525	2.357	.058	-0.369	[-0.750; 0.013]
ISI insomnia sum score (SR)	94	-2.741	0.922	.004 **	-0.455	[-0.760; -0.151]
RSES sum score (SR)	94	1.915	0.833	.025 *	0.283	[0.037; 0.530]
SCS total score (SR)	94	1.337	2.067	.519	0.087	[-0.180; 0.354]
BCSS negative-self (SR)	94	-0.423	0.735	.567	-0.077	[-0.341; 0.188]
BCSS positive-self (SR)	94	0.286	0.825	.730	0.055	[-0.258; 0.368]
BCSSS negative-others (SR)	94	-0.807	0.856	.348	-0.144	[-0.448; 0.160]
BCSS positive-others (SR)	94	0.053	0.813	.984	0.013	[-0.371; 0.396]

Notes: Regression analysis of the post-scores (dependent variable) on group (independent variable: 0 = WL, 1 = CBTd-E) while controlling for study site; PSYRATS delusions subscales printed in bold; * $p \leq .05$; ** $p \leq .01$; [1] = unstandardized beta; *SE* = Standard error; SR = Self-rating; PSYRATS = Psychotic Rating Symptom Scales; PDI = Peters et al. Delusions Inventory; EMA = ecological momentary assessment of persecutory delusions over 6 consecutive days and 10 time points: mean score; PANSS = Positive and Negative Syndrome Scale; PANSS POS = PANSS positive scale; PANSS NEG = PANSS negative scale; PANSS GEN = PANSS general psychopathology scale; CDSS = Calgary Depression Rating Scale; RFS = Role Functioning Scale; CPZ = chlorpromazine equivalent of antipsychotic medication; ERQ = Emotion Regulation Questionnaire; ERSQ = Emotion Regulation Style Questionnaire; ERI = Emotion Regulation Inventory; PSWQ = Penn State Worry Questionnaire; ISI = Insomnia Severity Index; RSE = Rosenberg Self-Esteem Scale; SCS = Self-Compassion Scale; BCSS = Brief Core Schema Scale.

Table S11: Results of the linear regression analysis with listwise deletion on the primary and secondary outcome variables and target mechanisms at 6-months post-assessment, including additional subscales of the PSYRATS delusions scale (blind assessment)

Measure	*n (analysis)*	Group differences *beta* unst.[1.]	*SE*	*p*	Effect size *d*	*95% CI*
Primary outcome variables						
PSYRATS delusions scale (Blind)	42	-2.145	2.056	.303	-0.451	[-1.327; 0.424]
PSYRATS delusions: amount of preoccupation	42	-0.044	0.330	.895	-0.041	[-0.670; 0.588]
PSYRATS delusions: duration of preoccupation	42	-0.871	0.384	.029*	-0.826	[-1.563; -0,089]
PSYRATS delusions: conviction	42	-0.163	0.498	.745	-0.138	[-0.995; 0.718]
PSYRATS delusions: amount of distress	42	-0.246	0.535	.648	-0.192	[-1.035; 0.652]
PSYRATS delusions: intensity of distress	42	-0.352	0.414	.401	-0.295	[-0.998; 0.408]
PSYRATS delusions: disruption of daily life	42	-0.343	0.300	.260	-0.407	[-1.127; 0.313]
PSYRATS delusions scale (Unblinded)	72	-2.236	1.488	.138	-0.635	[-1.479; 0.208]
Secondary outcome variables						
PDI-21 grand total score (SR)	62	-8.548	9.515	.373	-0.150	[-0.485; 0.185]
…EMA persecutory delusions (SR)	48	-0.154	0.249	.539	-0.110	[-0.470; 0.249]
PANSS POS (Blind)	47	-1.527	1.049	.153	-0.341	[-0.812; 0.131]
PANSS POS (Unblinded)	70	-1.760	0.872	.048 *	-0.470	[-0.936; -0.005]
PANSS NEG (Blind)	47	0.034	1.331	.980	0.007	[-0.582; 0.597]
PANSS NEG (Unblinded)	70	-0.536	1.014	.599	-0.112	[-0.537; 0.312]
PANSS GEN (Blind)	47	-3.755	1.888	.053	-0.558	[-1.124; 0.008]
PANSS GEN (Unblinded)	70	-3.018	1.593	.063	-0.468	[-0.962; 0.025]
CDSS total score (Blind)	47	-1.074	1.126	.345	-0.291	[-0.907; 0.325]
CDSS total score (Unblinded)	69	-2.301	0.729	.002 **	-0.540	[-0.881; -0.198]
RFS general functioning (Blind)	46	0.321	0.616	.605	0.147	[-0.422; 0.716]
RFS general functioning (Unblinded)	68	1.048	0.526	.051	0.411	[-0.001; 0.824]
RFS social functioning (Blind)	46	0.855	0.617	.173	0.391	[-0,178; 0.961]
RFS social functioning (Unblinded)	68	0.868	0.557	.124	0.372	[-0.105; 0.848]
CPZ	68	-123.283	171.588	.475	-0.245	[-0.928; 0.437]
PSYRATS voices scale (Blind)	42	-2.895	3.086	.354	-0.244	[-0.769; 0.282]
PSYRATS voices scale (Unblind)	72	-1.822	2.112	.391	-0.137	[-0.453; 0.180]
Target mechanisms						
ERQ expressive suppression (SR)	62	1.198	1.105	.283	0.219	[-0.186; 0.624]
ERQ reappraisal (SR)	61	4.371	1.452	.004 **	0.588	[0.197; 0.978]]

ERSQ total score (SR)	63	0.282	3.916	.943	0.016	[-0.428; 0.460]
ERI positive emotions (SR)	63	-3.557	1.977	.077	-0.352	[-0.744; 0.040]
ERI negative emotions (SR)	63	-2.541	2.893	.384	-0.232	[-0.762; 0.297]
PSWQ sum score (SR)	63	-6.556	3.488	.065	-0.520	[-1.075; 0.034]
ISI insomnia sum score (SR)	62	-3.074	1.304	.022 *	-0.493	[-0.911; -0.074]
RSES sum score (SR)	63	2.517	1.212	.042 *	0.364	[0.013; 0.715]
SCS total score (SR)	62	1.162	3.019	.702	0.073	[-0.308; 0.455]
BCSS negative-self (SR)	62	-0.073	1.069	.946	-0.013	[-0.391; 0.365]
BCSS positive-self (SR)	62	0.054	1.215	.964	0.010	[-0.443; 0.463]
BCSSS negative-others (SR)	61	-1.297	1.161	.269	-0.224	[-0.626; 0.178]
BCSS positive-others (SR)	62	-0.049	1.208	.968	-0.011	[-0.568; 0.546]

Notes: Regression analysis of the post-scores (dependent variable) on group (independent variable: 0 = WL, 1 = CBTd-E) while controlling for study site; PSYRATS delusions subscales printed in bold; * $p \leq .05$; ** $p \leq .01$; [1] = unstandardized beta; SE = Standard error; SR = Self-rating; PSYRATS = Psychotic Rating Symptom Scales; PDI = Peters et al. Delusions Inventory; EMA = ecological momentary assessment of persecutory delusions over 6 consecutive days and 10 time points: mean score; PANSS = Positive and Negative Syndrome Scale; PANSS POS = PANSS positive scale; PANSS NEG = PANSS negative scale; PANSS GEN = PANSS general psychopathology scale; CDSS = Calgary Depression Rating Scale; RFS = Role Functioning Scale; CPZ = chlorpromazine equivalent score of antipsychotic medication; ERQ = Emotion Regulation Questionnaire; ERSQ = Emotion Regulation Style Questionnaire; ERI = Emotion Regulation Inventory; PSWQ = Penn State Worry Questionnaire; ISI = Insomnia Severity Index; RSE = Rosenberg Self-Esteem Scale; SCS = Self-Compassion Scale; BCSS = Brief Core Schema Scale.

Table S12: Moderator analysis (hierarchical linear regression analysis): does the ability to regulate negative emotions moderate the association between intervention group (CBTd-E vs. Wait list) and delusions at 6-months post-assessment, controlling for the baseline scores of delusions and study site and using FIML as missing value strategy

Outcome variable	*n* (analysis)	*beta* $_{unst.}$ [1.]	*SE*	*p*	Effect size of the interaction: outcome x group *F2.*	*95% CI*
Primary outcome variables						
PSYRATS delusions scale (Blind)	58	-0.205	0.174	.239	0.04	[-0.547; 0.136]
PSYRATS delusions scale (Unblinded)	89	-0.171	0.139	.217	0.03	[-0.443; 0.100]
Secondary outcome variables						
PDI-21 grand total score (SR)	88	0.214	0.821	.795	0.00	[-1.396; 1.823]
EMA persecutory delusions (SR)	71	0.001	0.021	.969	0.00	[-0.041; 0.043]

Notes: [1] = unstandardized beta; SE = Standard error; SR = Self-rating; PSYRATS = Psychotic Rating Symptom Scales; PDI = Peters et al. Delusions Inventory; EMA = ecological momentary assessment of persecutory delusions over 6 consecutive days and 10 time points: mean score.

Table S13: Moderator analysis (hierarchical linear regression analysis) does the ability to regulate negative emotions moderate the association between intervention group (CBTd-E vs. Wait list) and delusions at 6-months post-assessment, controlling for the baseline scores of delusions and study site and using singe imputation as missing value strategy

Outcome variable	*n (analysis)*	*beta* unst.[1.]	*SE*	*p*	Effect size of the interaction: outcome x group *F2.*	*95% CI*
Primary outcome variables						
PSYRATS delusions scale (Blind)	94	-0.062	0.099	.535	0.00	[-0.259; 0.135]
PSYRATS delusions scale (Unblinded)	94	-0.096	0.114	.402	0.01	[-0.323; 0.131]
Secondary outcome variables						
PDI-21 grand total score (SR)	94	-0.193	0.658	.771	0.00	[-1.501; 1.116]
EMA persecutory delusions (SR)	94	0.006	0.014	.651	0.00	[-0.021; 0.034]

Notes: [1] = unstandardized beta; SE = Standard error; SR = Self-rating; PSYRATS = Psychotic Rating Symptom Scales; PDI = Peters et al. Delusions Inventory; EMA = ecological momentary assessment of persecutory delusions over 6 consecutive days and 10 time points: mean score.

Table S14: Moderator analysis (hierarchical linear regression analysis): does the ability to regulate negative emotions moderate the association between intervention group (CBTd-E vs. Wait list) and delusions at 6-months post-assessment, controlling for the baseline scores of delusions and study site and using listwise deletion as missing value strategy

Outcome variable	*n* (analysis)	*beta* unst.[1.]	*SE*	*p*	Effect size of the interaction: outcome x group *F2.*	*95% CI*
Primary outcome variables						
PSYRATS delusions scale (Blind)	41	-0.205	0.189	.284	0.03	[-0.588; 0.178]
PSYRATS delusions scale (Unblinded)	69	-0.171	0.146	.246	0.02	[-0.464; 0.121]
Secondary outcome variables						
PDI-21 grand total score (SR)	62	0.214	0.872	.807	0.00	[-1.534; 1.961]
EMA persecutory delusions (SR)	48	0.001	0.023	.971	0.00	[-0.045; 0.047]

Notes: [1] = unstandardized beta; SE = Standard error; SR = Self-rating; PSYRATS = Psychotic Rating Symptom Scales; PDI = Peters et al. Delusions Inventory; EMA = ecological momentary assessment of persecutory delusions over 6 consecutive days and 10 time points: mean score.

Table S15: Moderator analysis (hierarchical linear regression analysis): do negative self-schemata moderate the association between intervention group (CBTd-E vs. Wait list) and delusions at 6-months post-assessment, controlling for the baseline scores of delusions and study site and using FIML as missing value strategy

Outcome variable	*n (analysis)*	*beta* $_{unst.}$ [1.]	*SE*	*p*	Effect size of the interaction: outcome x group *F2.*	*95% CI*
Primary outcome variables						
PSYRATS delusions scale (Blind)	58	-0.215	0.344	.533	0.01	[-0.889; 0.460]
PSYRATS delusions scale (Unblinded)	88	-0.453	0.260	.081	0.02	[-0.963; 0.057]
Secondary outcome variables						
PDI-21 grand total score (SR)	87	-1.807	1.682	.282	0.02	[-5.103; 1.488]
EMA persecutory delusions (SR)	71	-0.017	0.042	.687	0.00	[-0.100; 0.066]

Notes: [1] = unstandardized beta; SE = Standard error; SR = Self-rating; PSYRATS = Psychotic Rating Symptom Scales; PDI = Peters et al. Delusions Inventory; EMA = ecological momentary assessment of persecutory delusions over 6 consecutive days and 10 time points: mean score.

Table S16: Moderator analysis (hierarchical linear regression analysis) do negative self-schemata moderate the association between intervention group (CBTd-E vs. Wait list) and delusions at 6-months post-assessment, controlling for the baseline scores of delusions and study site and using singe imputation as missing value strategy

Outcome variable	*n (analysis)*	*beta* $_{unst.}$ [1.]	*SE*	*p*	Effect size of the interaction: outcome x group *F2.*	*95% CI*
Primary outcome variables						
PSYRATS delusions scale (Blind)	94	-0.179	0.194	.358	0.01	[-0.564; 0.206]
PSYRATS delusions scale (Unblinded)	94	-0.404	0.213	.061	0.04	[-0.826; 0.019]
Secondary outcome variables						
PDI-21 grand total score (SR)	94	-0.193	0.658	.771	0.00	[-1.501; 1.116]
EMA persecutory delusions (SR)	94	-1.070	1.310	.416	0.01	[-3.673; 1.533]

Notes: [1] = unstandardized beta; SE = Standard error; SR = Self-rating; PSYRATS = Psychotic Rating Symptom Scales; PDI = Peters et al. Delusions Inventory; EMA = ecological momentary assessment of persecutory delusions over 6 consecutive days and 10 time points: mean score.

Table S17: Moderator analysis (hierarchical linear regression analysis): do negative self-schemata moderate the association between intervention group (CBTd-E vs. Wait list) and delusions at 6-months post-assessment, controlling for the baseline scores of delusions and study site and using listwise deletion as missing value strategy

Outcome variable	*n* *(analysis)*	*beta* $_{unst.}$[1.]	*SE*	*p*	Effect size of the interaction: outcome x group *F2.*	*95% CI*
Primary outcome variables						
PSYRATS delusions scale (Blind)	41	-0.215	0.372	.568	0.01	[-0.971; 0.541]
PSYRATS delusions scale (Unblinded)	68	-0.453	0.275	.104	0.04	[-1.002; 0.096]
Secondary outcome variables						
PDI-21 grand total score (SR)	62	-1.807	1.785	.316	0.02	[-5.385; 1.771]
EMA persecutory delusions (SR)	48	-0.017	0.045	.709	0.00	[-0.108; 0.074]

Notes: [1] = unstandardized beta; SE = Standard error; SR = Self-rating; PSYRATS = Psychotic Rating Symptom Scales; PDI = Peters et al. Delusions Inventory; EMA = ecological momentary assessment of persecutory delusions over 6 consecutive days and 10 time points: mean score.

Table S18: Therapist competency rated with the Cognitive Therapy Rating Scale Revised for Psychosis in *n* = 110 audios.

CTS-RP	*M*	*SD*
Agenda setting	4.2	1.1
Feedback	4.6	0.8
Collaboration	4.7	0.8
Effective time use	4.8	0.8
Positive focus	4.4	1.0
Therapeutic relationship	4.8	0.5
Assessing key emotions	4.2	1.2
Eliciting key cognitions	4.3	1.1
Eliciting key behaviours	4.2	1.2
Guided discovery	4.3	1.0
Application of change methods (CBTd-E techniques)	4.5	0.9
Homework	4.1	1.1
General therapist competency	4.2	1.1
Total score	49.0	10.3

References

Nittel, C. M., Lincoln, T. M., Lamster, F., Leube, D., Rief, W., Kircher, T., & Mehl, S. (2018). Expressive suppression is associated with state paranoia in psychosis. An experience sampling study on the association between adaptive and maladaptive emotion regulation strategies and paranoia. *British Journal of Clinical Psychology 57*(3), 291–312. https://doi.org/https://doi.org/10.1111/bjc.12174

Schlier, B., Moritz, S., & Lincoln, T. M. (2016). Measuring fluctuations in paranoia: Validity and psychometric properties of brief state versions of the Paranoia Checklist. *Psychiatry Res*, *241*, 323–332. https://doi.org/10.1016/j.psychres.2016.05.002
